# Stoichiometric constraints on the microbial processing of carbon with soil depth along a riparian hillslope

**DOI:** 10.1007/s00374-018-1317-2

**Published:** 2018-10-10

**Authors:** Laura L. de Sosa, Helen C. Glanville, Miles R. Marshall, Andrea Schnepf, David M. Cooper, Paul W. Hill, Andrew Binley, Davey L. Jones

**Affiliations:** 1 Environment Centre Wales, Bangor University, Deiniol Road, Bangor, Gwynedd LL57 2UW UK; 2 School of Geography, Geology and the Environment, Keele University, Keele, Staffordshire ST5 5BG UK; 3 Department of Forest and Soil Sciences, University of Natural Resources and Applied Life Sciences, Vienna, Austria; 4 Centre for Ecology and Hydrology, Environment Centre Wales, Deiniol Road, Bangor, Gwynedd LL57 2UW UK; 5 Lancaster Environment Centre, Lancaster University, Lancaster, LA1 4YQ UK; 6 UWA School of Agriculture and Environment, The University of Western Australia, 35 Stirling Highway, Crawley, WA 6009 Australia

**Keywords:** Recalcitrant carbon, Nitrogen, Phosphorus, Nutrient cycling, Subsoil

## Abstract

Soil organic matter (SOM) content is a key indicator of riparian soil functioning and in the provision of ecosystem services such as water retention, flood alleviation, pollutant attenuation and carbon (C) sequestration for climate change mitigation. Here, we studied the importance of microbial biomass and nutrient availability in regulating SOM turnover rates. C stabilisation in soil is expected to vary both vertically, down the soil profile and laterally across the riparian zone. In this study, we evaluated the influence of five factors on C mineralisation (C_min_): (i) substrate quantity, (ii) substrate quality, (iii) nutrient (C, N and P) stoichiometry, (iv) soil microbial activity with proximity to the river (2 to 75 m) and (v) as a function of soil depth (0–3 m). Substrate quality, quantity and nutrient stoichiometry were evaluated using high and low molecular weight ^14^C-labelled dissolved organic (DOC) along with different nutrient additions. Differences in soil microbial activity with proximity to the river and soil depth were assessed by comparing initial (immediate) C_min_ rates and cumulative C mineralised at the end of the incubation period. Overall, microbial biomass C (MBC), organic matter (OM) and soil moisture content (MC) proved to be the major factors controlling rates of C_min_ at depth. Differences in the immediate and medium-term response (42 days) of C_min_ suggested that microbial growth increased and carbon use efficiency (CUE) decreased down the soil profile. Inorganic N and/or P availability had little or no effect on C_min_ suggesting that microbial community growth and activity is predominantly C limited. Similarly, proximity to the watercourse also had relatively little effect on C_min_. This work challenges current theories suggesting that areas adjacent to watercourse process C differently from upslope areas. In contrast, our results suggest that substrate quality and microbial biomass are more important in regulating C processing rates rather than proximity to a river.

**Electronic supplementary material:**

The online version of this article (10.1007/s00374-018-1317-2) contains supplementary material, which is available to authorized users.

## Introduction

Agricultural grasslands represent one of the biggest managed stores of carbon (C) in the terrestrial biosphere (Jones and Donnelly 2004). Further, it is widely accepted that soil organic C (SOC) underpins a range of regulating, provisioning, cultural and supporting ecosystem services in these habitats (Adhikari and Hartemink 2016). It is therefore vital that we preserve SOC levels in grassland landscapes to ensure continual delivery of these services. However, this requires a good understanding of the factors that regulate C turnover and to identify what management practices promote greater SOC retention.

Whilst below-ground respiration represents a good general indicator of SOC turnover, it provides little indication as to whether the C is of plant or microbial origin and from where within the soil profile the CO_2_ originates (Robert 2002; van Hees et al. 2005; Rui et al. 2016). Recent research suggests that C dynamics differ through the soil profile and, albeit controversial, the processes regulating C storage in topsoils and subsoils may be different (Salome et al. 2010; Sanaullah et al. 2011). Some authors have suggested that different microbial patterns at depth are due to a decrease in substrate quality (more recalcitrant and less biodegradable) and are thus only able to support small, specialist microbial populations (Rovira and Vallejo 2002; Salome et al. 2010). Other authors support the idea that subsoil microbial communities are more C efficient due to a permanent limitation of available substrate (Fierer et al. 2003; Blagodatskaya et al. 2007). Studies comparing C responses within the soil profile, however, have often found contradictory results. For example, C addition has been shown to induce both positive and negative priming of native SOC (Kuzyakov 2002; Zhang et al. 2015; Wordell-Dietrich et al. 2017). This highlights our lack of knowledge about how, and to what extent, differences in microbial composition, substrate quality and also microbial activity influence C and nutrient turnover within the soil profile.

The availability of inorganic nutrients (e.g. N, P, S) in soil has also been shown to be a key factor regulating rates of SOC turnover (Creamer et al. 2016). In this context, fertiliser addition to grasslands can be expected to significantly alter the ratio of C to other essential nutrients (nutrient stoichiometry). If the stoichiometry (e.g. C:N:P ratio) approaches the optimal ratio required for microbial cells, and there are no other limiting factors (e.g. pH, water, oxygen availability), then microbial growth will occur leading to C storage (Cleveland and Liptzin 2007; Fierer et al. 2003; Sinsabaugh et al. 2013). As the stoichiometry of microbial groups in soils is different (e.g. fungi versus bacteria), the microbial response to fertiliser addition may differ both horizontally and vertically (topsoil vs. subsoil), due to heterogeneous and localised shifts in microbial community composition.

The transitional area between aquatic and terrestrial ecosystems (e.g. riparian areas) are thought to play a key role in SOC decomposition due to having potentially greater microbial specialisation which has evolved in response to high-frequency disturbance regimes such as, fluctuations of aerobic/anaerobic conditions (Gregory et al. 1991; Clinton et al. 2002; Lewis et al. 2003). Additionally, flood pulses spreading out across the riparian zone have been shown to be the precursor for intermittent cycles of organic matter (OM) accumulation or abrupt removal (Acuna et al. 2004; Naiman et al. 2010) therefore, these areas are expected to have higher respiration rates due to microbial communities responding rapidly to environmental conditions (Tufekcioglu et al. 1998).

Within the context of a grassland riparian transect, the main objectives of our study were: (1) to test how nutrient (C, N and P) quantity and stoichiometry affects the rate of C mineralisation (C_min_) down the soil profile; (2) to explore how substrate quality and stoichiometry affects the turnover of both low and high molecular weight (MW) DOC; (3) to assess the influence of soil depth (0–3 m) on rates of C_min_; and (4) to evaluate the influence of proximity (2–75 m) to the river on C turnover rates. We hypothesised that nutrient limitation would be a greater constraint to C turnover in subsoils relative to topsoils and that this would be most apparent for labile forms of C which should drive faster microbial growth. We also hypothesised that C turnover would be greatest closest to the river due to it being a zone of higher nutrient enrichment.

## Materials and methods

### Study site

The area of study is located within the Conwy Catchment, North Wales (UK) (53° 12′ 5.33′′ N 3° 46′ 54.66′′ W) (Fig. S1). A detailed description of the catchment can be found in Emmett et al. (2016), Sharps et al. (2017) and de Sosa et al. (2017). The experimental site comprised a 3 ha typical improved grassland hillslope (mean slope of 20%) used for intensive livestock (sheep and cattle) production. The soil is free draining and classified as a Eutric Endoleptic Cambisol (WRB 2014) and the dominant vegetation consists of *Lolium perenne* L. and *Trifolium repens* L. The mean annual rainfall is 1230 mm (based on 30-year average 1961–1990 data from the UK Met Office) and the mean annual temperature (at 30 cm depth) is 8 °C (based on 30-year average 1981–2010 data from the UK Met Office).

### Soil core sampling

**Fig. 1:**
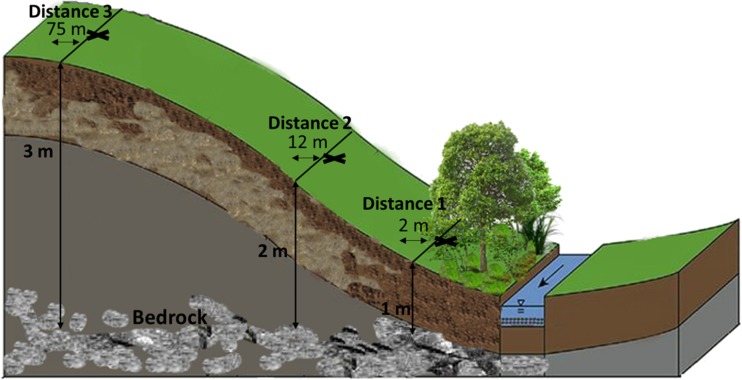
Location of sample points across the riparian hillslope. Horizontal arrows indicate distance from the river (these are referred in the manuscript to as distance 1, 2 and 3). Vertical arrows indicate the total length of extractable core, determined according to the maximum depth of the soil profile (presence of bedrock) or until an impermeable (e.g. clay layer) boundary as determined by a geophysical survey (Fig. S2) was reached

Three 75 m long transects, 20 m apart, were identified across the hillslope, running perpendicular to the river (Fig. 1). Along each transect, intact soil cores were extracted at 2, 12 and 75 m (from this point onwards in the manuscript, these are referred to as distance 1, 2 and 3, respectively) using a Cobra percussion hammer corer (Van Walt Ltd., Haslemere, Surrey, UK) in May 2016. The total length of extractable core was determined according to the maximum depth of the soil profile (presence of bedrock) or until an impermeable (e.g. clay layer) boundary as determined by a geophysical survey (Fig. S2-S3) was reached (distance 1 = 1 m total core length, distance 2 = 2 m total core length and distance 3 = 3 m total core length; *n* = 18 × individual 1 m core lengths). Intact soil cores were extracted in 1 m lengths (4 cm diameter; total cores *n* = 18) and wrapped in thin-walled polyethylene (PE) sleeves to maintain core integrity and immediately transferred to the laboratory and stored at 4 °C prior to analysis.

### Geophysical survey

Electrical geophysical surveys were carried out in order to assess major lithological units at the site. Six parallel 94 m long transects of 48 electrodes were used with a Syscal Pro (Iris Instruments, Orleans, France) to perform electrical resistivity tomography (ERT) surveys. A dipole-dipole electrode configuration (Binley 2015) was used to maximise sensitivity to lateral variability at the site. ERT data were modelled using the R2 inverse code (http://www.es.lancs.ac.uk/people/amb/Freeware/R2/R2.htm) to produce 2D vertical sections of resistivity to a maximum soil depth of 10 m.

### General soil characterisation

Soil cores were divided into depth intervals of 0–15, 15–30, 50–100, 100–150, 150–200 and 250–300 cm (from this point onwards in the manuscript, these are grouped and referred to as topsoil (0–30 cm), midsoil (50–100 cm) and deepsoil (100–300 cm), respectively), and sieved (< 5 mm) in order to remove stones and any plant material and to ensure sample homogeneity. This mesh size was chosen as it minimises changes in microbial activity (Jones and Willet 2006). Soil moisture content (MC) was determined gravimetrically (24 h, 105 °C) and soil organic matter content (SOM) was determined by loss-on-ignition (LOI) (16 h, 450 °C). Soil pH and electrical conductivity (EC) were measured using standard electrodes in a 1:2.5 (*w*/*v*) soil-to-deionised water mixture. Exchangeable ammonium (NH_4_-N) and nitrate (NO_3_-N) in soil were determined with 0.5 M K_2_SO_4_ extracts (1:5 *w*/*v*) via the colorimetric procedure of Mulvaney (1996) and the vanadate method of Miranda et al. (2001), respectively. Phosphate was quantified with 0.5 M acetic acid extracts (1:5 *w*/*v*; Fisher et al. 1998) following the ascorbic acid-molybdate blue method of Murphy and Riley (1962) and total C (TC) and N (TN) were determined with a TruSpec® elemental analyser (Leco Corp., St Joseph, MI). Dissolved organic C (DOC) and total dissolved N (TDN) were quantified in 1:5 (*w*/*v*) soil-to-0.5 M K_2_SO_4_ extracts using a Multi N/C 2100 TOC analyser (AnalytikJena, Jena, Germany). Microbial biomass-C (MBC) was measured using the fumigation-extraction method (Vance et al. 1987) after 72 h of fumigation (*k*_*ec*_ = 0.45 and *k*_*en*_ = 0.54). Samples were analysed for phospholipid fatty acid (PLFA) concentration according to the 96-well format, high throughput method of Buyer and Sasser (2012) (Microbial ID Inc., Newark, DE). Sorption of NH_4_^+^ was assessed as described by Marsden et al. (2016). In brief, six concentrations ranging from 5 to 200 mg NH_4_-N l^−1^ in 0.01 M CaCl_2_ were added to 0.5 g of field moist soil (1:5 *w*/*v* soil-to-extractant ratio) and shaken for 0.5 h at 150 rev min^−1^ on a rotary shaker. Subsequently, an aliquot (1.5 ml) was centrifuged (10,000*g*; 5 min) and the supernatant analysed as described above. The total amount of NH_4_^+^ adsorbed was determined by the difference between the initial amount of NH_4_-N added and the final remaining in solution. Any NH_4_^+^ not recovered in the solution was assumed to be adsorbed onto the solid phase or taken up by microbial cells. Phosphorus (P) sorption was determined following an adapted method of Nair et al. (1984). In brief, 1.0 g field moist soil was shaken in 0.01 M CaCl_2_ (1:25 *w*/*v* soil-to-extractant ratio) containing known concentrations of P (0, 0.5, 1, 5, 10, 50 mg P l^−1^ as Na_2_HPO_4_) spiked with ^33^P (0.06 kBq ml^−1^; PerkinElmer Inc., Waltham, MA) to determine the amount of P adsorbed onto the solid phase. These concentrations were selected due to their likelihood of being encountered in natural systems. Samples were shaken for 2 h (150 rev min^−1^, 25 °C) on an orbital shaker. This time was chosen in order to assess intermediate equilibrium conditions (respective equilibrium time established in Santos et al. 2011). After 2 h, 1.5 ml of supernatant was removed and centrifuged (10,000*g*, 5 min). Subsequently, 1 ml of supernatant was mixed with 4 ml of Optiphase HiSafe 3 liquid scintillation fluid (PerkinElmer Inc.) and the amount of ^33^P activity remaining in solution measured using a Wallac 1404 liquid scintillation counter (Wallac EG & G, Milton Keynes, UK). The total amount of P adsorbed was determined by the difference between the initial ^33^P activity added and the final amount of ^33^P remaining in solution. Any P not recovered in the solution was assumed to be sorbed onto the soil’s solid phase. To estimate the soil absorption maxima of P, sorption isotherms were examined according to the linearised form of the Langmuir equation (Reddy and Kadlec 1999; Mehdi et al. 2007).

### Preparation of nutrient solutions

To investigate how nutrient stoichiometry affected C mineralisation (C_min_) rates, soil samples collected from the hillslope were incubated with N, P and N + P together, in combination with three different C amendments, namely:

1. High dose of low MW DOC
2. Low (natural abundance) dose of low MW DOC
3. Medium (natural abundance) dose of high MW DOC

We tested four different nutrient additions for each C amendment

1. C only addition (C)
2. C and N addition (CN)
3. C, N and P addition (CNP), and
4. C and P addition (CP)

C, N and P treatments were added in mass ratios of C:*N* = 9 (N in the form of NH_4_NO_3_) and C:*P* = 85 (P in the form of Na_2_HPO_4_) to represent the average stoichiometric ratios of the soil microbial biomass in grassland systems (Cleveland and Liptzin 2007).

The different C amendments were chosen to simulate distinct soil C conditions within the soil. For the high dose C experiment, 300 mM C (specific C addition of 36 μg C g^−1^ dry soil) was chosen to represent soil C released during root cell lysis and would likely stimulate microbial growth (Jones and Darrah 1996; Tabuchi et al. 2004). For the low (natural abundance) C experiment, a total of 6 μM C (specific C addition of 0.72 ng C g^−1^ dry soil) was added to simulate the background C concentrations found under natural conditions (Boddy et al. 2007). Glucose was selected as a labile source of low MW DOC for the low and high (hotspot) conditions as it represents a common root exudate dominating the low MW DOC pool and is known to be important in soil C cycling (van Hees et al. 2005). It is also capable of being assimilated by almost all soil microorganisms. For the high MW C experiment, 47.4 mM of high MW (> 1 kDa) recalcitrant DOC (specific addition of 18.2 μg C g^−1^ dry soil) was selected to represent the compounds remaining once the labile fractions have been utilised by microbial populations (Gillis and Price 2016). This concentration is at the high end of the range reported for soil solutions from grassland soils (Christou et al. 2005). The recalcitrant DOC was obtained following the incubation and subsequent decomposition of ^14^C-labelled *Calluna vulgaris* (L.) Hull. shoots in a Sapric Histosol for 2 years. Soil pore water was recovered using Rhizon® samplers (Rhizosphere Research Products B.V., Wageningen, The Netherlands) (Jones et al. 2015).

### Preparation of isotopically labelled solutions

Nutrient solutions, as described above, were spiked with uniformly ^14^C-labelled D-glucose (PerkinElmer Inc.) for the high and low C dosages only. For both C doses, the specific activity added was 0.2 kBq ml^−1^. The concentration of ^14^C added (< 10 nM) did not significantly alter the C concentration of the unlabelled (^12^C) nutrient solutions. For the high MW DOC, nutrient solutions were spiked with ^14^C-labelled DOC (specific activity 0.07 kBq ml^−1^). To ensure the plant-derived DOC solution was only composed of high MW material, the solution was purified using an Amicon 8050 stirred cell equipped with a 1 kDa ultrafiltration membrane (Millipore UK Ltd., Hertfordshire, UK).

### Carbon mineralisation

To measure the rate of ^14^C-substrate mineralisation, 5 g soil (dry weight equivalent to account for soil water content variability down the soil profile) were placed into sterile 50 ml polypropylene tubes. To determine the rate of ^14^CO_2_ evolution, 50 μl of ^14^C-glucose-labelled nutrient solution for the low and high C treatments, and 160 μl of the high MW ^14^C-DOC-labelled nutrient solution (higher volume used to account for the lower specific activity of this solution) were added to the soil surface. Immediately after nutrient addition, a 5 cm^3^ polypropylene vial containing NaOH (1 ml, 1 M) was added into the tubes to capture any evolved ^14^CO_2_. The tubes were hermetically sealed and incubated at 10 °C to represent the mean annual temperature of the catchment. The NaOH traps were changed after 0.5, 1, 2, 4, 6, 24, 48, 72, 96, 120, 144, 168, 192 and 336 h and then weekly up to 6 weeks after initial ^14^C-labelling for both glucose-C additions. For the high MW DOC experiment, traps were changed at 1, 6, 24, 48, 72, 168, 336, 504, 672, 840, 1176, 1512 and 1680 h due to the slower mineralisation rates. On removal, the NaOH traps were mixed with Optiphase HiSafe 3® liquid scintillation fluid (PerkinElmer Inc.) and the amount of ^14^CO_2_ captured determined using a Wallac 1404 liquid scintillation counter (Wallac EG & G).

### Data and statistical analysis

To assess if C dynamics were regulated by different microbial mechanism with depth and with distance from the river, initial (immediate) C_min_ rates and total C mineralised at the end of the incubation period were calculated for all treatments and C amendments. The specific initial C_min_ rates were calculated for a 6-h incubation period or when the linear phase was achieved for the experiments involving the low and high doses of ^14^C-glucose (low MW DOC) and for 72 h for the high MW recalcitrant DOC. An *r*^2^ value of > 0.90 was deemed an acceptable cut-off value for assessing linearity rates (number of observations = 504). Due to large differences in microbial biomass down the soil profile, C_min_ rates results were normalised according to biomass size (i.e. C_min_ rates/MBC). Both the normalised, and the actual respiration rates per soil unit are reported. For data normalisation, MBC was chosen over PLFA biomass due to low percentages of biomarkers found down at depth in the PLFA analysis.

Total C mineralised was calculated as the C cumulative percentage of evolved ^14^CO_2_ recovered at the end of the incubation period respective to the amount of C added at the beginning of the experiment.

Statistical analysis was performed with SPSS version 22 for Windows (IBM Corp., Armonk, NY) and R (R Core Team 2012). All data were assessed for normality and homogeneity of variance with Shapiro Wilk’s tests and Levene’s statistics, respectively. Transformations to accomplish normality were done when necessary (log_10_-transformed variables: nitrate content, available P, DOC, TDN). For all statistical tests, *P* < 0.05 was selected as the significance cut-off value. Separate analysis of variance (one-way ANOVA) tests were performed to explore differences in soil physicochemical properties with respect to: (1) distance from the river, followed by Tukey’s post-hoc test, and (2) depth, followed by Games-Howell post-hoc test; this test was selected due to not achieving homoscedasticity of variables with depth as a factor and Games-Howell is more robust in this respect. A principal component analysis (PCA) was used to explore the spatial (depth and distance) relationships of soil physicochemical properties. Effects of depth, distance from the river, and treatment on mineralisation were tested using a mixed-effects model with depth, distance and treatment as fixed effects and transects as random effects. Interactions between variables were included for each model when a significant improvement of the model (*P* < 0.05) was observed. A significant improvement in the model was tested by performing an analysis of variance (ANOVA) of the full model both with, and without, inclusion of the effect being tested. Both *F* and *P* values are reported to assess variability between groups. Differences in soil depth, nutrient treatment, and distance from the river were tested with Tukey post-hoc tests. Visual inspection of residual plots did not reveal any obvious deviations from homoscedasticity or normality. To assess if different soil properties might be useful predictors of soil C_min_, a step-wise multiple regression was conducted analysing relationships between C_min_ rates, final percentage respired and specific soil properties.

## Results

### Soil physicochemical properties

**Fig. 2:**
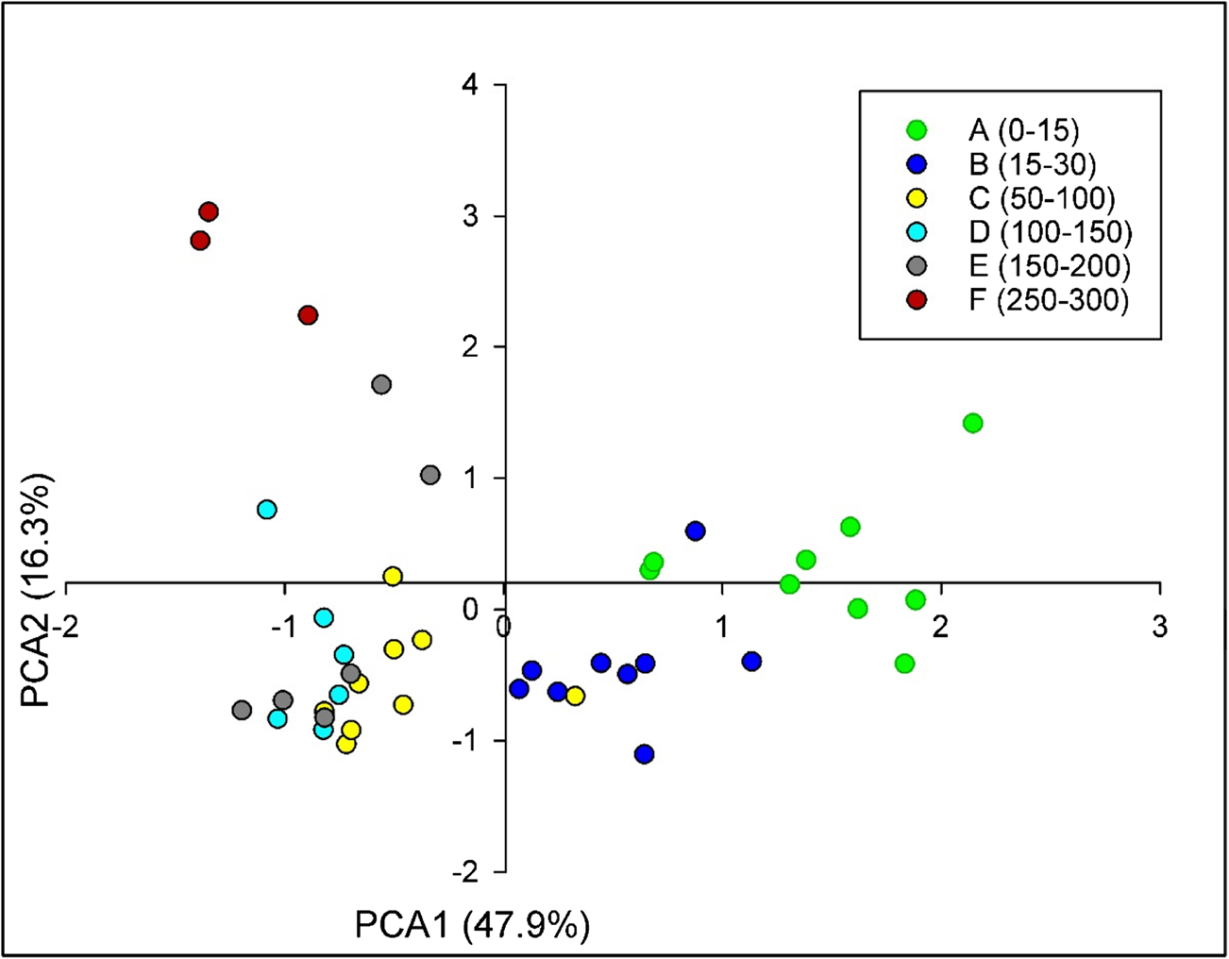
Correlation bi-plot from the principal component analysis (PCA) on soil physicochemical variables across the hillslope (*n* = 9 for A, B, C; *n* = 6 for D, E; *n* = 3 for F). Correlation of soil properties with the main axes is given by arrows and sample points by colour dots. Nitrogen adsorption (Nads). Phosphorus maxima adsorption (Pmax). Ratio carbon (C)/ nitrogen (N) (C:N). Moisture content (MC). Electrical conductivity (EC)

Principal component analysis (PCA) of all the soil physicochemical variables across the hillslope (*n* = 42, irrespective of distance or depth) identified two principal components (PC) which together, explain 64.2% of the total variance within the dataset (Fig. 2). Organic matter content, exchangeable NH_4_^+^-N, maximum P adsorption (Pmax) and C:N ratio correlated significantly (*P* < 0.05) with PC1, whilst available P, N adsorption (Nads), pH and EC correlated significantly (*P* < 0.05) with both PC1 and PC2. The spatial segregation of samples within the PCA revealed the strong effect of depth on physicochemical properties irrespective of distance from the river. However, some physicochemical properties differed (*P* < 0.05) according to proximity to the watercourse, but only within certain sampling depths. The topsoil (0–30 cm) for distance 3 showed an increase of almost a third in OM content compared to distance 1. Similarly, DOC was 2 times greater at distance 3 in comparison with distance 1 for topsoil. The midsoil depth (50–100 cm), again for distance 3, showed higher, more alkaline, pH values compared to distances 1 and 2. NH_4_^+^-N tended to increase by almost 4 times with distance from the river for the topsoil whereas for the mid- and deepsoil zones a fourfold higher NH_4_^+^-N content was found in areas closest to the river (Table S1). P adsorption maxima increased on average by 29% and 37% from distance 1 to distance 2 and 3 respectively for the top 15 cm whereas it was 25% and 34% greater from distance 1 to distance 2 and 3 at 15–30 cm sampling depth (Table S2).

With respect to depth, a decrease of most physicochemical properties was identified, except for pH and available P (Table S1). Amongst all physicochemical properties, MC, OM, DOC, TDN and microbial biomass-C displayed the greatest differences from top soil to deepsoil for all distances.

### Geophysical survey

Similar geophysical patterns were observed along the six independent transects (Fig. S3). In the upper part of each transect a low resistivity zone is noted at 2 to 3 m depth. We attribute this to a dense clay-rich unit. In the lower part of the transect a distinct resistive zone can be seen at a depth of ~ 4 m, which is likely to be the soil-bedrock interface.

### High dose of low MW DOC addition to soil

#### Total C mineralised

On average, the total percentage of C mineralised was 40.7% ± 0.9 irrespective of distance from the river, depth or nutrient treatment. Overall, the total percentage of C mineralised was higher in deeper layers than in the topsoil (Table 1) but was not affected by nutrient treatment (*P* > 0.05). After 42 days of incubation, the total C mineralised increased by 36.8% and 26.8% from the top layer to the midsoil and deepsoil (250–300 cm) respectively, and irrespective of nutrient treatment and distance from the river (Table 1). The total amount of C mineralised was affected by the proximity to the river and the treatment added but only distance 3 was significantly different from the other two (*P* < 0.001). Overall, distance 3 mineralised lower amounts of C for all treatments and depths (Table 1). Particularly at a sampling depth of 50–100 cm, the amount of C mineralised was on average 35% higher at distance 1 than distance 3. This effect was especially noticeable for the C-only treatment (Table 1) which could be due to the inherent nutrient variability within distances (Table S1).

**Table 1:** Total ^14^CO_2_ production following the addition of a high dose of low molecular weight ^14^C-DOC to soil either in the presence or absence of nutrients (N and/or P) as a function of soil depth and distance from the river. Soils were incubated with the ^14^C-labelled substrate for 42 days. The ANOVA results (F and *P* value) are shown for a mixed effects model with depth, distance from the river and treatment as fixed effects and transect as a random effect. Interactions were only included when a significant improvement (*P* > 0.05, italics) of the model fit was observed. Values are means ± standard errors (*n* = 3). Missing values indicate no samples due to hitting bedrock

Total ^14^CO_2_ (% of total ^14^C added)	Distance from the river	Soil depth																	
		0–15 cm			15–30 cm			50–100 cm			100–150 cm			150–200 cm			250–300 cm		
DOC only	2 m	35.7	±	3.9	41.0	±	0.5	59.5	±	1.4		–			–			–	
	12 m	33.2	±	1.3	38.0	±	0.5	46.3	±	7.2	49.8	±	3.4	46.3	±	6.8		–	
	75 m	30.5	±	0.8	36.0	±	0.6	24.3	±	13	39.9	±	6.2	28.1	±	9.4	43.4	±	2.0
DOC + N	2 m	32.7	±	3.4	36.9	±	1.4	54.3	±	3.4		–			–			–	
	12 m	34.3	±	3.4	37.7	±	1.7	46.6	±	5.8	47.6	±	2.6	49.8	±	5.4		–	
	75 m	31.0	±	0.7	43.2	±	7.4	45.1	±	6.6	52.1	±	2.0	51.5	±	0.8	51.6	±	0.7
DOC + N + P	2 m	39.3	±	5.2	38.3	±	1.7	56.6	±	4.7		–			–			–	
	12 m	29.2	±	1.6	42.3	±	4.5	50.2	±	3.2	49.1	±	1.5	49.8	±	6.2		–	
	75 m	28.7	±	0.5	33.0	±	0.2	45.6	±	3.9	46.3	±	2.9	50.4	±	3.8	51.4	±	0.4
DOC + P	2 m	31.2	±	4.1	39.2	±	1.5	56.9	±	3.7								–	
	12 m	30.1	±	0.6	45.3	±	4.0	46.7	±	4.1	51.8	±	3.1	43.2	±	6.2		–	
	75 m	29.7	±	0.5	31.2	±	3.0	31.2	±	11	31.8	±	4.8	35.8	±	7.4	42.2	±	7.0
ANOVA results	Soil depth			Distance from the river			Nutrient treatment				Soil depth * Nutrient treatment					Distance * Nutrient treatment			
	F	*P* value		F	*P* value		F			*P* value	F		*P* value			F	*P* value		
	21.33	**<** *0.001*		21.52	**<** *0.001*		2.17			0.09	–		–			2.98	*0.008*		

### Initial C mineralisation rates

**Fig. 3:**
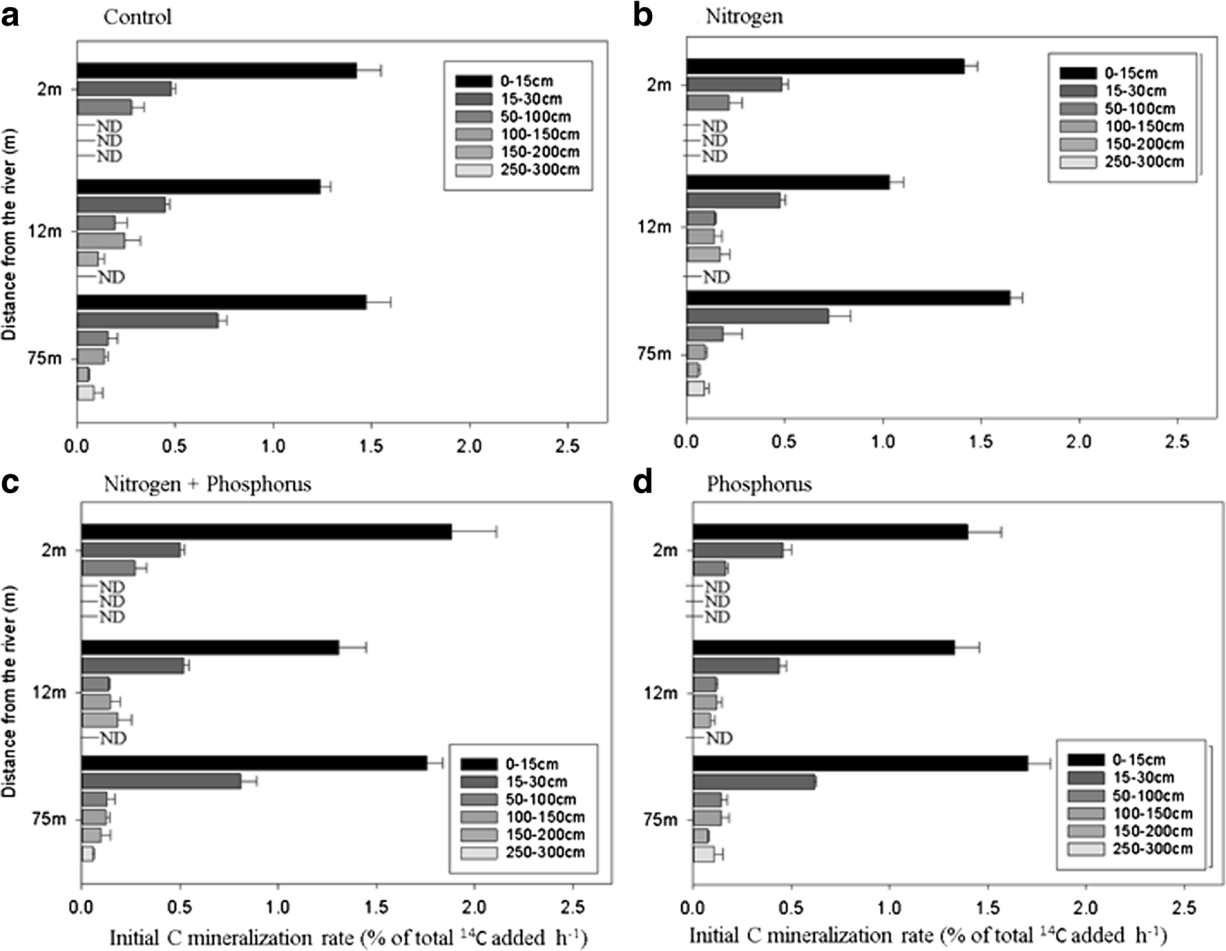
Initial C mineralisation rates measured during the initial linear phase (between 0 and 6 h) after the addition of a high dose of low molecular weight DOC either alone or in combination with N, P or N + P. Values are presented for three different distances from the river (2, 12 and 75 m) and for 6 different soil depths. Soil depths were grouped into topsoil (0–30 cm), midsoil (50–100 cm) and deepsoil (100–300) in the manuscript for the description of the factors assessed. Bars represent mean values (*n* = 3) ± standard errors. ND equates to no data due to hitting bedrock (Fig. 1)

Soil depth was the main factor controlling C_min_ rates regardless of treatments (Table 2). Overall, C_min_ rates significantly decreased from the topsoil (*P* < 0.001) down to 100 cm whereas no significant effects (*P* > 0.05) were identified below that depth (Fig. 3). Regardless of treatment or distance, the amount of C evolved (relative to the % of total ^14^C added) decreased by 82 and 88% from the topsoil to the midsoil and deepsoil depths, respectively (Fig. 3). A lag phase of about 4 days corresponding to microbial growth was displayed in some sampling depths below 50 cm after the addition of C and/or nutrients whereas no such effect was observed above 50 cm (Fig. S4). The effect of distance from the river also affected C_min_ rates but only distances 2 and 3 were significantly different from each other (*P* < 0.001). The addition of N or P both separately and combined had little or no effect on C_min_ rates irrespective to the distance from the river and depth (*P* > 0.05). The multiple regression analysis (data not shown) identified MBC, OM and MC as the best predictors explaining C_min_ rates. Significant positive correlations were found between C_min_ rates and the aforementioned physicochemical properties (*r*^2^ > 0.69 ± 0.01 for MC, *r*^2^ > 0.83 ± 0.01 for OM and *r*^2^ > 0.81 ± 0.02 for MBC, *P* < 0.001 in all cases) irrespective of the treatment.

**Table 2:** Results of ANOVA (F and *P* value) for the mixed effects model with soil depth, distance from the river and nutrient treatment as fixed effects, transect as a random effect and initial C mineralisation rate as the independent variable. Interactions were only included when a significant improvement (*P* > 0.05, italics) of the model fit was observed. High and low doses of labile dissolved organic carbon (DOC) refer to the amounts of low MW C added to the soil in the experiment (see section 2.4)

ANOVA results	Soil depth		Distance from the river	Nutrient treatment			Depth * nutrient treatment		Row * Nutrient treatment	
	F	*P* value	F	*P* value	F	*P* value	F	*P* value	F	*P* value
High dose of low MW labile DOC	395	**<** *0.001*	8.92	**<** *0.001*	2.39	0.07	–	–	–	**–**
Low dose of low MW labile DOC	178	**<** *0.001*	21.0	**<** *0.001*	2.82	*0.04*	1.87	*0.03*	–	**–**
High MW recalcitrant DOC	57.3	**<** *0.001*	10.5	**<** *0.001*	3.69	*0.01*	11.0	**<** *0.001*	4.73	**<** *0.001*

Due to large differences in total microbial biomass within the soil profile, results were normalised by the MBC data in order to identify different trends in SOM decomposition (% of the total added ^14^C mg^−1^ biomass C h^−1^). Neither treatment, distance, nor depth had a significant effect on C_min_ rates (Fig. S5). Furthermore, no interactions between the fixed effects were found.

### Low dose of low MW DOC addition to soil

#### Total C mineralised

After 42 days of incubation, 30.4% ± 0.5 of the added C was mineralised regardless of distance, depth and nutrient treatment (Table 3). The total amount of C mineralised generally increased with depth for the N and P treatments (*P* < 0.001) whereas the control (C only addition) showed a decrease of 18% from topsoil (0–15 cm) to the deepsoil layer in distance 3 (Table 3). The overall effect of treatment increased with depth (*P* < 0.001). From the topsoil to the deepsoil, total C mineralised increased by 30%, 25 and 24% (relative to the initial % of ^14^C added) for N, NP and P treatments, respectively. However, although NP and P-only treatment were different from the control (*P* < 0.001) they did not differ from each other.

**Table 3:** Total ^14^CO_2_ production following the addition of a low dose of low molecular weight ^14^C-DOC to soil either in the presence or absence of inorganic nutrients (N and/or P) as a function of soil depth and distance from the river. Soils were incubated with the ^14^C-labelled substrate for 42 days. The ANOVA results (F and *P* value) are shown for a mixed effects model with depth, distance from the river and treatment as fixed effects and transect as a random effect. Interactions were only included when a significant improvement (*P* > 0.05, italics) of the model fit was observed. Values are means ± standard errors (*n* = 3). Missing values indicate no samples due to hitting bedrock

Total ^14^CO_2_ (% of total ^14^C added)	Distance from the river	Soil depth								
		0–15 cm		15–30 cm	50–100 cm		100–150 cm	150–200 cm	250–300 cm	
DOC only	2 m	26.5 ± 0.3		25.7 ± 1.2	30.8 ± 3.5		–	–	–	
	12 m	26.1 ± 2.2		26.1 ± 0.3	28.9 ± 3.5		23.0 ± 3.9	28.1 ± 2.1	–	
	75 m	23.4 ± 3.5		26.8 ± 0.7	29.6 ± 2.4		28.4 ± 0.2	24.9 ± 5.6	19.1 ± 6.0	
DOC + N	2 m	31.5 ± 1.2		34.4 ± 5.5	37.1 ± 1.8		–	–	–	
	12 m	29.2 ± 0.4		29.1 ± 2.2	30.3 ± 3.5		34.4 ± 1.2	41.5 ± 1.6	–	
	75 m	29.6 ± 0.6		28.8 ± 0.5	33.5 ± 2.6		35.3 ± 1.2	32.0 ± 5.1	41.2 ± 0.0	
DOC + N + P	2 m	30.6 ± 0.6		29.5 ± 0.9	32.5 ± 1.8		–	–	–	
	12 m	27.9 ± 0.7		27.8 ± 0.9	29.5 ± 1.6		30.2 ± 1.3	37.7 ± 1.1	–	
	75 m	27.8 ± 0.5		27.8 ± 2.0	24.5 ± 4.4		32.5 ± 2.0	33.6 ± 0.2	36.3 ± 2.7	
DOC + P	2 m	27.7 ± 1.5		27.7 ± 0.7	29.1 ± 0.7		–	–	–	
	12 m	33.8 ± 4.7		27.2 ± 1.8	27.9 ± 1.4		25.9 ± 0.8	37.2 ± 1.8	–	
	75 m	25.4 ± 0.9		32.3 ± 6.7	27.2 ± 1.7		32.2 ± 2.2	40.6 ± 0.8	37.3 ± 0.3	
ANOVA results	Soil depth			Distance from the river	Nutrient treatment		Soil depth * Nutrient treatment		Distance * Nutrient treatment	
	F	*P* value	F	*P* value	F	*P* value	F	*P* value	F	*P* value
	12.08	**<** *0.001*	2.66	0.07	35.66	**<** *0.001*	3.95	**<** *0.001*	–	**–**

#### Initial C mineralisation rates

**Fig. 4:**
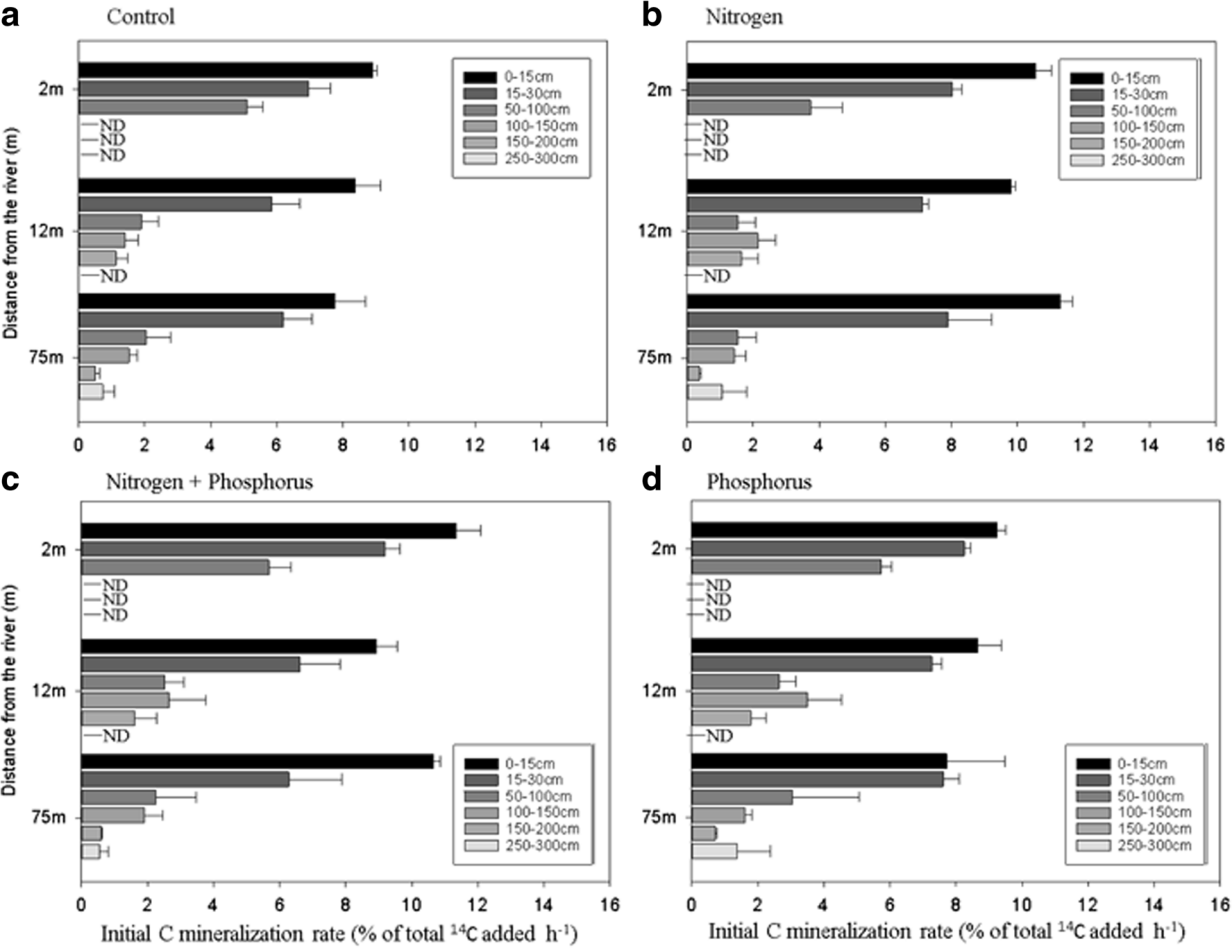
Initial C mineralisation rates measured during the initial linear phase (between 0 and 6 h) after the application of a low dose of low molecular weight DOC either alone or in combination with N, P or N + P. Values are presented for three different distances from the river (2, 12 and 75 m) and for 6 different soil depths. Soil depths were grouped into topsoil (0–30 cm), midsoil (50–100 cm) and deepsoil (100–300) in the manuscript for the description of the factors assessed. Bars represent mean values (*n* = 3) ± standard errors. ND equates to no data due to hitting bedrock (Fig. 1)

The initial rates of C_min_ were strongly influenced by depth (*P* < 0.001, Table 2) ranging from 9.43% ± 0.27 in the topsoil to 0.93% ± 0.29 of the total ^14^C added h^−1^ for the deepsoil depth, irrespective of nutrient treatment and distance from the river (Fig. 4). However, significant differences were only identified within depth intervals between 0 and 100 cm, whilst between 100 and 300 cm, no differences were apparent. Nutrient treatment also showed an effect on C_min_ rates although this effect was influenced by depth as the interaction (*P* = 0.04). Across the full range of sampling depths, C_min_ was 3 times greater in the top layer than the midsoil for the control and 5, 4 and 2 times greater for the CN, CNP and CP treatments respectively. Carbon mineralisation rates in the deepsoil were almost 8 times lower than the top layer for the control and N addition treatments but only 5 times lower for the treatment with P alone. Distance from the river also influenced C_min_ rates but only the distance closest to the river was different compared to the other two distances (*P* < 0.001). In particular, the midsoil showed on average, and irrespective of nutrient treatment, 50% faster C_min_ rates compared with the other two distances (Fig. 4). Rates of C_min_ were strongly correlated with MC (*r*^2^ = 0.74 ± 0.02), OM (*r*^2^ = 0.68 ± 0.05) and MBC (*r*^2^ = 0.61 ± 0.04) (*P* < 0.001 in all cases) irrespective of the treatment.

As with the high rate of low MW DOC addition, nutrient treatment showed no effect on C_min_ rates after adding a low dose of DOC when the data was normalised to MBC (*P* > 0.05). However, the effect of distance and depth still had an overall significant effect on C_min_ rates (*P* < 0.001) (Fig. S6).

### Medium dose of high MW DOC addition to soil

#### Total C mineralised

Overall, the total amount of C mineralised was 11.7% ± 0.6 regardless of distance, depth and treatment after 70 days of incubation (Table 4). In general, total C_min_ decreased with depth up to 100 cm, below which the total C remained relatively consistent regardless of nutrient treatment or distance from the river. However, a significant effect of treatment with respect to depth was identified (*P* < 0.001). The addition of P-only decreased C_min_ fivefold in distance 1 and by twofold in distance 2 in the topsoil in comparison with the rest of the treatments (Table 4).

**Table 4:** Total ^14^CO_2_ production following the addition of a medium dose of high molecular weight ^14^C-DOC to soil either in the presence or absence of inorganic nutrients (N and/or P) as a function of soil depth and distance from the river. Soils were incubated with the ^14^C-labelled substrate for 70 days. The ANOVA results (F and *P* value) are shown for a mixed effects model with depth, distance from the river and treatment as fixed effects and transect as a random effect. Interactions were only included when a significant improvement (*P* > 0.05, italics) of the model fit was observed. Values are means ± standard errors (*n* = 3). Missing values indicate no samples due to hitting bedrock

Total ^14^CO_2_ (% of total ^14^C added)	Distance from the river	Soil depth																	
		0–15 cm			15–30 cm			50–100 cm			100–150 cm			150–200 cm			250–300 cm		
DOC only	2 m	26.1	±	1.5	18.8	±	0.4	9.8	±	2.6		–			–			–	
	12 m	25.8	±	5.0	16.8	±	0.5	7.1	±	2.0	6.1	±	1.5	4.6	±	0.6		–	
	75 m	27.8	±	5.1	21.4	±	3.0	4.7	±	1.0	4.7	±	1.8	3.9	±	0.4	3.9	±	0.2
DOC + N	2 m	23.6	±	1.4	18.5	±	0.3	8.1	±	1.2		–			–			–	
	12 m	17.3	±	2.0	16.2	±	0.2	10.0	±	2.3	5.4	±	0.5	4.8	±	0.5		–	
	75 m	23.5	±	1.8	19.5	±	0.7	4.8	±	1.1	5.4	±	0.5	3.4	±	0.1	3.2	±	0.0
DOC + N + P	2 m	22.1	±	0.8	16.6	±	0.9	8.8	±	1.9		–			–			–	
	12 m	16.5	±	1.2	14.5	±	0.5	7.8	±	0.6	5.8	±	1.1	4.6	±	0.5		–	
	75 m	20.7	±	1.5	16.8	±	1.3	3.9	±	1.1	4.2	±	1.2	3.6	±	0.1	3.4	±	0.4
DOC + P	2 m	4.6	±	1.1	22.8	±	4.3	17.6	±	3.0		–			–			–	
	12 m	9.2	±	3.3	16.5	±	0.9	22.1	±	1.1	5.0	±	0.4	4.3	±	0.3		–	
	75 m	15.2	±	4.1	21.0	±	2.3	12.5	±	6.9	3.9	±	0.3	3.3	±	0.1	3.5	±	0.3
ANOVA results	Soil depth				Distance from the river			Nutrient treatment			Soil depth * Nutrient treatment						Distance * Nutrient treatment		
	F	*P* value	F		*P* value			F	*P* value			F	*P* value		F		*P* value		
	98.91	**<** *0.001*	0.97		0.38			1.81	0.14			11.16	**<** *0.001*		–		**–**		

#### Initial C mineralisation rates

**Fig. 5:**
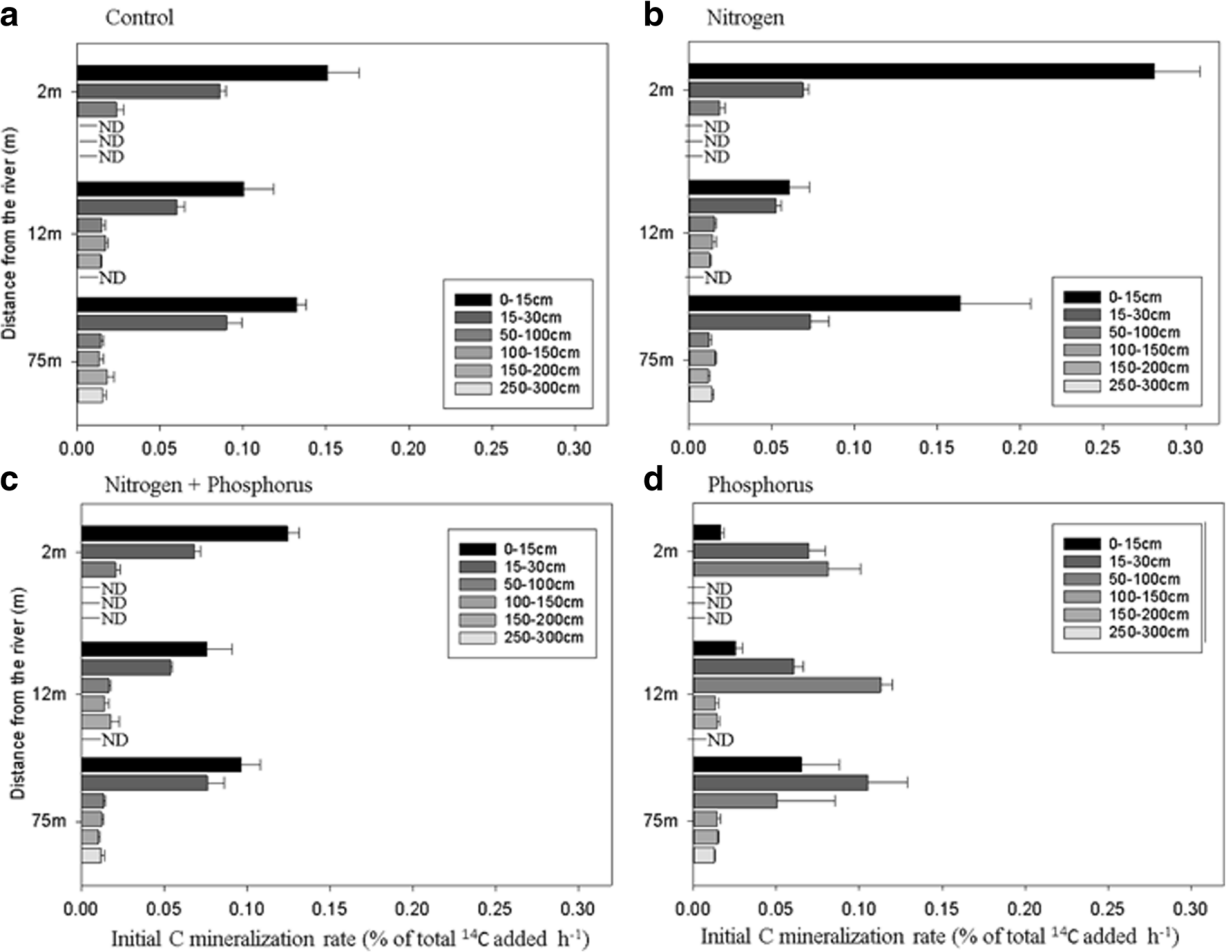
Initial C mineralisation rates measured during the initial linear phase (between 0 and 48 h) after the application of a medium dose of high MW DOC either alone or in combination with N, P or N + P. Values are presented for three different distances from the river (2, 12 and 75 m) and for 6 different soil depths. Soil depths were grouped into topsoil (0–30 cm), midsoil (50–100 cm) and deepsoil (100–300) in the manuscript for the description of the factors assessed. Bars represent mean values (*n* = 3) ± standard errors. ND equates to no data due to hitting bedrock (Fig. 1)

The high MW DOC was mineralised at a maximum rate of 0.28% h^−1^. This rate of mineralisation was 85% and 97% slower rate than for the high and low labile C additions respectively after 72 h across all depth, treatments and distances (Table 2, Fig. 5). Topsoil displayed, on average, 6.5 times greater C_min_ rates than deeper layers (> 50 cm) for the control, N and NP treatments irrespective of distance. However, the P-only treatment resulted in a decrease of 30% in C_min_ rates from topsoil to deepsoil layers, although this effect was particularly notable at distances 1 and 2 (Fig. 5). Regarding the treatment effect in the topsoil, the addition of P alone or in combination with N caused a decrease in C_min_ rates of 93 and 33% compared to the control and N alone treatments respectively. Distance from the river also caused different responses in C_min_ rates (*P* < 0.001) but this effect was mainly evident within the top layer and in response to the addition of P which appeared to have a repressive effect on C_min_. As for the previous C amendments, MC, OM and MBC (*r*^2^ < 0.65 ± 0.02, *r*^2^ < 0.76 ± 0.01, *r*^2^ < 0.63 ± 0.04 respectively, *P* < 0.001 in all cases) explained a large part of C_min_ variability for all treatments except for the P-only addition which only correlated with available P (*r*^2^ = 0.34, *P* < 0.05). Values of C_min_ rates normalised by the MBC showed no effect with distance or depth (Fig. S7; *P* < 0.05). Additionally, nutrient treatment also influenced C depletion but only the P addition treatment was different from the other three.

## Discussion

### Effect of soil depth and substrate quantity on C mineralisation

Soil depth had the most striking effect on C_min_ irrespective of the amount, or type, of C added or the incubation time. The fact that microbial communities are regulated by different controlling factors and nutrient limitations at depth has been endorsed before by the few studies that have explored C dynamics at depth (Fierer et al. 2003; Tian et al. 2017). Salome et al. (2010) identified greater spatial heterogeneity in soil physicochemical properties at depth and Manzoni et al. (2012) and Rey et al. (2005) have indicated that soil moisture also represents an important constraint on C turnover. Work presented by van Hees et al. (2005) and references therein, reported similar decomposition percentages as found in this study whilst Heitkötter et al. (2017a, b) indicated major differences in microbial C demand at different soil depths. Our results support these findings over the full duration of our experiment. Both high and low C additions showed faster decomposition rates in the topsoil compared to the deepsoil during the first hours of the experiment which is in good agreement with Rey et al. (2005) and Sanaullah et al. (2011). Some argue that this effect could be due to a more active microbial community in response to regular C (rhizodeposition) and nutrient inputs (N_2_ fixation and fertilisers) in grassland systems (Fontaine et al. 2003; Treseder 2008). However, it is worth noting that although the topsoil in our study was initially more responsive to the labile low MW source of C, the size of the microbial population, which was highly correlated to C_min_ rates, was on average 87-fold greater in the top layer compared to the deepest layers (Table S1). Therefore, when C_min_ rates are expressed on a per unit MBC basis (Fig. S5-S7), a much faster use of C was seen at depth irrespective of the source of C (relative to the low biomass at depth). Fierer et al. (2003) described the opposite pattern in respiration rates, however, their results were normalised by water potential and temperature relative to soil depth. Zhang et al. (2016) found the same negative correlation between PLFA biomass and moisture content as this study and also described a major shift in the depth pattern for soil respiration when it was normalised for microbial biomass.

We also observed that the addition of the high dose of C induced microbial growth (indicated by an initial lag phase in the mineralisation profile, Fig. S4) in the midsoil and deepsoil; a trend also identified by other authors (Blagodatskaya et al. 2014; Sanaullah et al. 2011). This growth pattern is related to the higher amount of C being added relative to the amount of microbial biomass-C with increasing depth.

Interestingly, even though the topsoil had an initially faster mineralisation rate in response to labile C addition, we observed higher amounts of C mineralised in deeper layers than in topsoil at the end of the experiment. This suggests a higher overall usage of the substrate for catabolic processes by the microbial community from deep soils (i.e. lower C use efficiency). Heitkötter et al. (2017a, b) found that C_min_ also increased with depth and Kemmitt et al. (2008) indicated that C_min_ at depth were independent of microbial biomass (much lower at depth in our study as shown in Table S1). In addition, it also indicated that the real limiting step for C_min_ was regulation by abiotic processes (e.g. chemical oxidation or hydrolysis, desorption from the solid phase, diffusion from inaccessible soil pores) that allowed the conversion of non-bioavailable humified soil OM into bioavailable OM. Therefore, results from this study indicate that once the substrate reaches microbial communities at depth, and this is bioavailable, they respond more rapidly and efficiently than in the topsoil.

Our results are opposite to that of Heitkötter et al. (2017a, b) where a higher total amount of C mineralised, in the form of organic acids, was reported for the topsoil. However, from our study, and others, there is evidence that supports the hypothesis that microbial communities have different substrate preferences and nutrient limitations which may control both degradation rate and microbial C use efficiency (Chen et al. 2012; Don et al. 2017; Fontaine et al. 2007).

Contrastingly, the addition of the high MW C source caused a noticeable decrease in C_min_ at depth. We hypothesised that the low bioavailability of the substrate would result in enhanced C storage rather than mineralisation at depth, due to the limited microbial populations not being able to obtain enough C and energy necessary for enzyme production and microbial growth required to breakdown the more recalcitrant compounds. In addition, there is also the possibility that some of the high MW C source may become unavailable (through association with mineral surfaces or, spatial isolation within soil aggregates) for microbial degradation. In this sense, the presence of a Fe-rich clay layer identified at depth (Table S3) supports this theory (Allison 1973; Bergaya and Lagaly 2006; Jastrow et al. 2007; Oades 1988). In contrast, we assume that being uncharged, the biodegradation of the low MW C substrate (glucose) will not be impeded by interaction with mineral surfaces (i.e. it will have a faster diffusion in soil and will not become trapped in aggregates).

### Effect of nutrient addition on C mineralisation

Studies on C_min_ rates currently show a wide disparity in response to nutrient addition. For example, in some cases a priming of SOM decomposition may occur after the addition of nutrients, whilst in others a negative, or no, effect has been reported (Conde et al. 2005; Janssens et al. 2010; Liljeroth et al. 1994; van Hees et al. 2005). In our study, the addition of nutrients (N and P) had no immediate or long-term effect when high amounts of C were supplied (Fig. 3). This lack of an overall effect suggests that soil microbial communities were severely C limited. Therefore, in our study we conclude that microbial mineralisation was driven by the microbial need for C rather than for N or P (Heuck et al. 2015).

However, under low inputs of labile C (background C content), greater CO_2_ fluxes (both initial and total) were observed after N and NP addition (Fig. 4), particularly for the top and midsoil, indicating greater nutrient limitation than in deeper layers and also a change in nutrient stoichiometry (C:N:P) (Fig. 4). This fact could reflect a more active and abundant microbial community whose maintenance requirements are higher due to their adaptation to a permanent supply of available substrate and therefore more C is used for respiration (Fontaine et al. 2003; Paterson et al. 2009; Treseder 2008; van Bodegom 2007).

Regarding the high high MW C treatment, the addition of nutrients had minimal or no effect on C turnover suggesting that this is not a preferred C substrate and that the community at depth has not adapted to using this chemically complex form of C. Interestingly, the addition of P together with the high MW C treatment had a suppressive effect on C_min_ rates in the topsoil, especially for distances 1 and 2 (Fig. 5). Bauhus and Khanna (1994) found a similar response on C depletion after the addition of P and Amador and Jones (1995) reported a lack of effect, or a depression, on C_min_ rates. Although this effect has been rarely explored, it has been attributed to differences in P and organic C concentrations, P adsorption capacities, changes in soil acidity or even toxicity of P for the soil biota (Bauhus and Khanna 1994; Kelly and Henderson 1978; Keller et al. 2006; Kelly and Nömmik 1978; Peng and Thomas 2010). In our study, the lack of any effect with P addition with the labile C source and the high intrinsic soil P concentration, as well as the reduction of P adsorption capacities (Table S1-S2), suggest that there was no toxic effect and no P limitation in areas close to the river. Therefore, we hypothesised that the non-preferential nature of the high MW C (judging by the small percentage respired) and the high P concentration in areas close to river, could have led to a decrease in the microbial C:N:P ratio up to such a point that this ratio was not optimum to induce microbial substrate decomposition. However, further work should be conducted to gain further insight on this inhibition.

### Effect of substrate quality on C mineralisation

The quality of the C source (meaning susceptibility to microbial enzyme degradation) has also been identified as an important driver controlling mineralisation rates (Bölscher et al. 2016; Chen et al. 2014; Rui et al. 2016; Shahbaz et al., 2017). The use of complex and low-quality substrates requires high activation energies (extracellular enzymes) (Bosatta and Ågren 1999) and because of this, a very low percentage of the high MW C added was used for microbial respiration (Fig. 5). Mechanistically, this suggests that although decomposers are able to break down the recalcitrant SOC, the energy gained is lower than the energy needed to catabolise such substrate and therefore long-term storage is preferred (Fontaine et al. 2007).

### Effect of distance from the river on C mineralisation

Areas adjacent to watercourse are assumed to play a key role in C dynamics mainly due to the influence of hydrologic regimes and riparian vegetation which: 1) controls import/export OM fluxes between the watercourse and the floodplain, 2) creates fluctuations of anaerobic/aerobic conditions regulating C source/sink balance, and 3) encourages more diverse microbial communities (Camino-Serrano et al. 2016; Gurtz et al. 1988; King et al. 2016; Lewis et al. 2003). However, as far as soil physicochemical properties are concerned, our results disagree with the general assumption of more potential for C storage within the riparian zone. Stutter et al. (2012) indicated a greater OM content in areas close to the river whereas we found less. Nevertheless, these areas corresponded to unmanaged vegetated buffer strips, mostly fenced and subject to agricultural use. In our case, the first sampling distance from the edge of the river (2 m) fell outside of this very narrow vegetated buffer strip, preventing us therefore from seeing if any difference existed. In support of our findings, Giese et al. (2000) also could not establish a relationship between percent C in the soil and distance from the main channel across the riparian transect.

However, we did identify some interesting patterns which suggest different microbial responses with respect to distance from the river. It should be noted that although the statistical analysis showed a significant effect with respect to distance (i.e. distance from the river) across the full range of C and nutrients amendments, it cannot be assumed that this effect reflects the influence of the riparian zone. Thus, the addition of a high dose of labile low MW C exhibited differences in C_min_ rates between the areas more distal to the river (distance 2 and 3) suggesting it is more related to the inherent physicochemical spatial variability rather the influence of the riparian zone. Similarly, for the high MW treatment, an effect of distance from the river was also displayed. However, this effect was more related to the suppressive effect of P on C_min_ (see section above) rather than the influence of the riparian zone.

We consistently detected faster C turnover at the midsoil depth after the addition of low MW C (i.e. treatments showed little or no effect). Wilson et al. (2011) illustrated the importance of flooding for C dynamics and microbial community composition. Our results suggest that this soil layer which was highly connected to fluctuating hydrology and nutrients, may have developed a more diverse microbial population although the present study only assessed the composition of main soil microbial groups (Naiman and Decamps 1997). However, this highlights that further research is needed to explore the role microbial diversity plays in riparian areas; currently, most riparian research targets specific processes rather than microbial communities of interest (Chen et al. 2012; Gutknecht et al. 2006; Seitzinger 1994).

## Conclusions

Global warming and the increases in CO_2_ emissions from land use change and fossil fuel burning could considerably influence SOM residency time (i.e. increase root exudation and microbial activity). Results from our study revealed higher decomposition potential within the deepsoil depth after labile low MW substrate addition, even though the top 15 cm exhibited faster immediate decomposition rates which might indicate different microbial C use efficiencies down the soil profile. Nutrient addition had little or no effect on C_min_ suggesting that overall the soil microbial community was C limited. Therefore, fast cycling of SOM is likely to occur in subsoil if any change in land use or agricultural management increases the input of labile C down the soil profile. Using a more recalcitrant, high MW source of C, we show that different C processing mechanisms were activated in the topsoil and deepsoil. Whereas a slow-cycling C decomposition prevailed in the topsoil, microbial mineralisation in the deepsoil was much slower which supports previous studies showing that microbial substrate preferences and nutrient limitation control the speed of degradation. In our study, the effect of the proximity to the river was minimal for all treatments within the experiment. Whilst this study has provided information underpinning C dynamics through the soil profile, which is important for managerial and modelling future scenarios, e.g. land use change, however, further work is required to investigate the links between soil microbial diversity and functioning (e.g. by determining gene expression) as a function of depth.

## Electronic supplementary material

ESM 1 (DOCX 7652 kb)
