# Supplementary material for: Stoichiometric constraints on the microbial processing of carbon with soil depth along a riparian hillslope
Source: Biol Fertil Soils. 2018 Oct 10;54(8):949–63. doi: 10.1007/s00374-018-1317-2 (PMC6413827; doi:10.1007/s00374-018-1317-2)
Supplement: Supplementary file 1 — (DOCX 7652 kb) [file 374_2018_1317_MOESM1_ESM.docx]

**Stoichiometric constraints on the microbial processing of carbon with soil depth along a riparian hillslope**

Laura L. de Sosa^a,*^, Helen C. Glanville^a,c^, Miles R. Marshall^a^, Andrea Schnepf^d^, David M. Cooper^b^ Paul W. Hill^a^, Andrew Binley^e^, Davey L. Jones^a,f^

^a^ *Environment Centre Wales, Bangor University, Deiniol Road, Bangor, Gwynedd, LL57 2UW, United Kingdom*

^b^ *Centre for Ecology and Hydrology, Environment Centre Wales, Deiniol Road, Bangor, Gwynedd, LL57 2UW, United Kingdom*

^c^ *School of Geography, Geology and the Environment, Keele University, Keele, Staffordshire, ST5 5BG, United Kingdom*

^d^ *Department of Forest and Soil Sciences University of Natural Resources and Applied Life Sciences, Vienna, Austria*

^e^ *Lancaster Environment Centre, Lancaster University, Lancaster, LA1 4YQ, United Kingdom*

^f^ *UWA School of Agriculture and Environment, The University of Western Australia, 35 Stirling Highway, Crawley, WA 6009, Australia*

* Corresponding author. Environment Centre Wales, Bangor University, Deiniol Road, Bangor, Gwynedd, LL57 2UW, United Kingdom.

*E-mail address*: [afs411@bangor.ac.uk](mailto:afs411@bangor.ac.uk) (L.L. de Sosa).

**Supplementary on-line information**

**
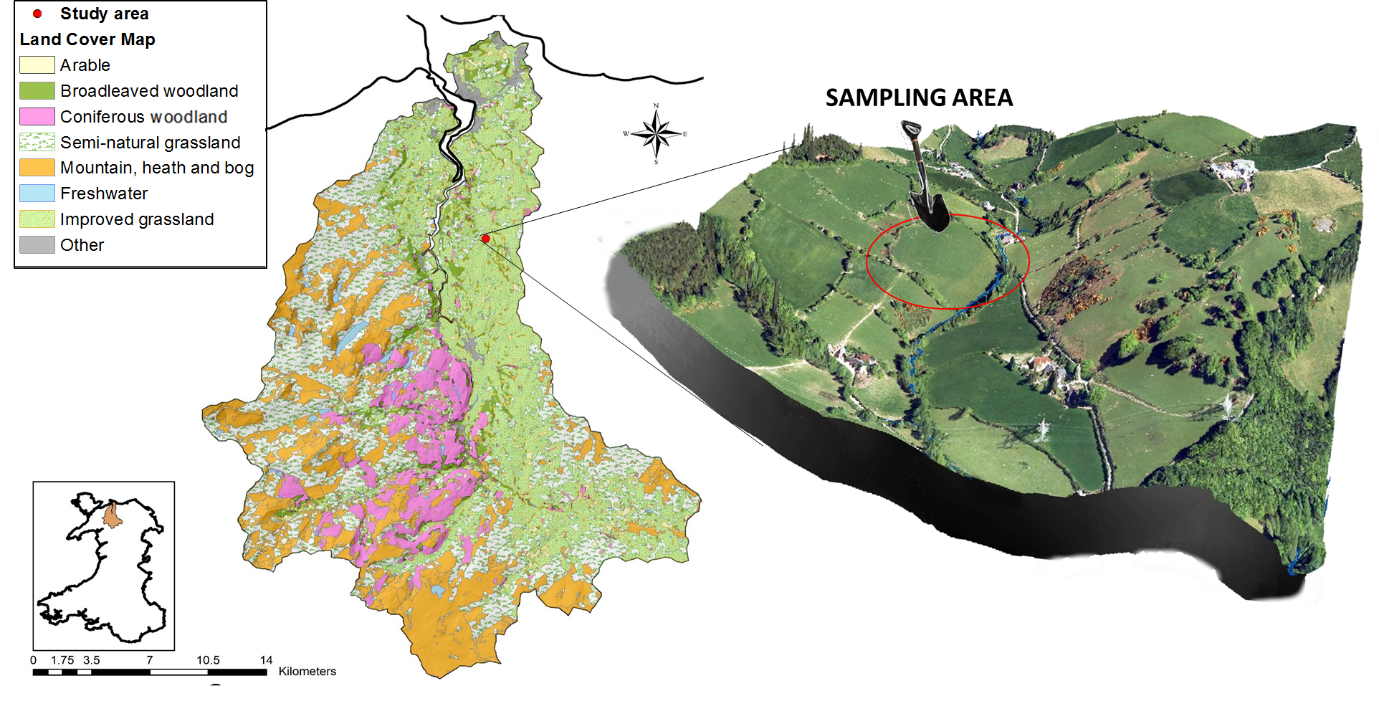
**

**Figure S1.** The Conwy catchment, North Wales, UK showing the location of the riparian sampling area and the major land cover classes according to Phase 1 classification.

**Geophysical survey**


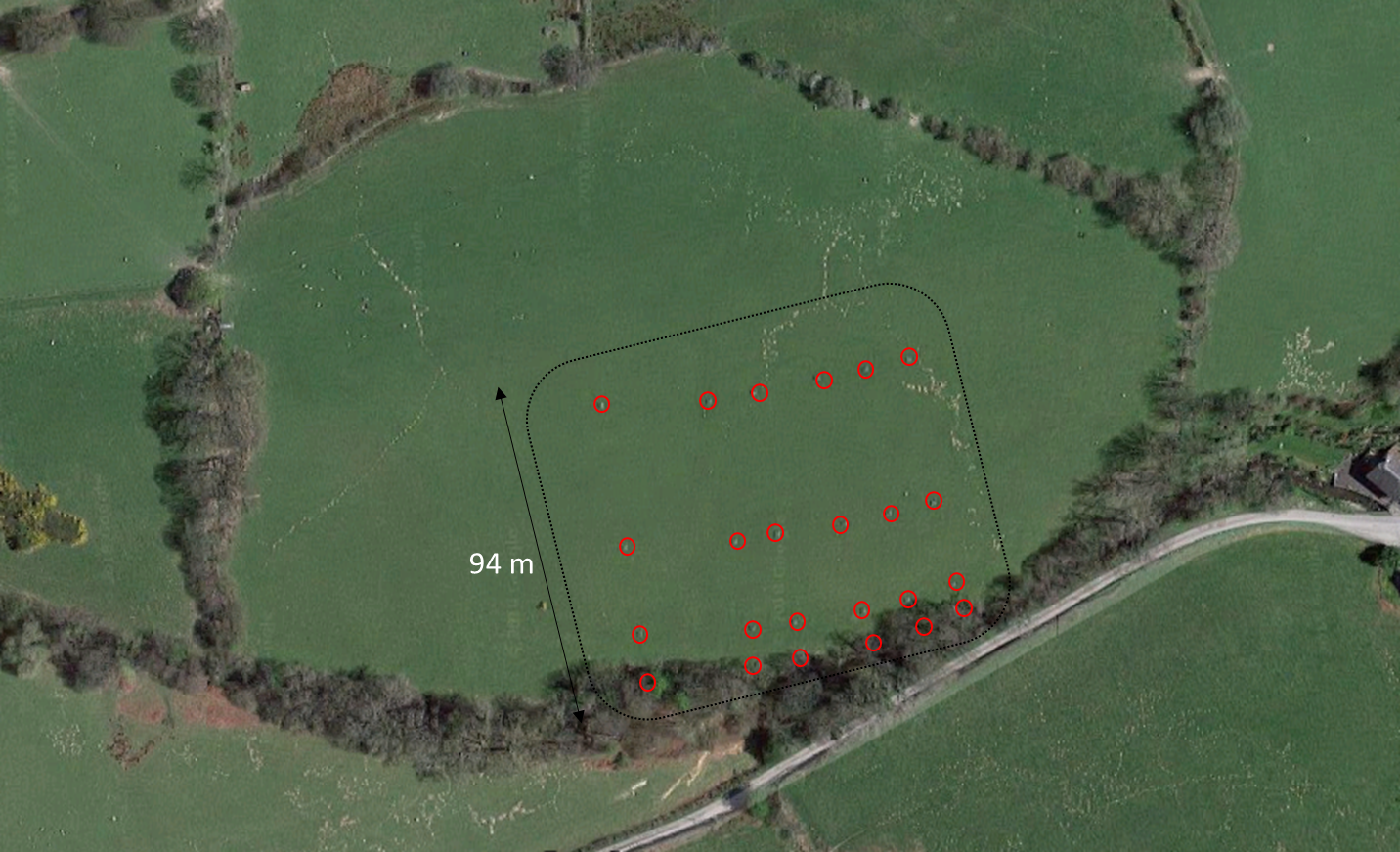


**Fig. S2.** Location of cages (red dots, *n* = 24) used to delineate the total area (dashed black box) for the geophysical survey across the hillslope.

**Fig. S3.** Ground conductivity data acquired across the area of study.

**
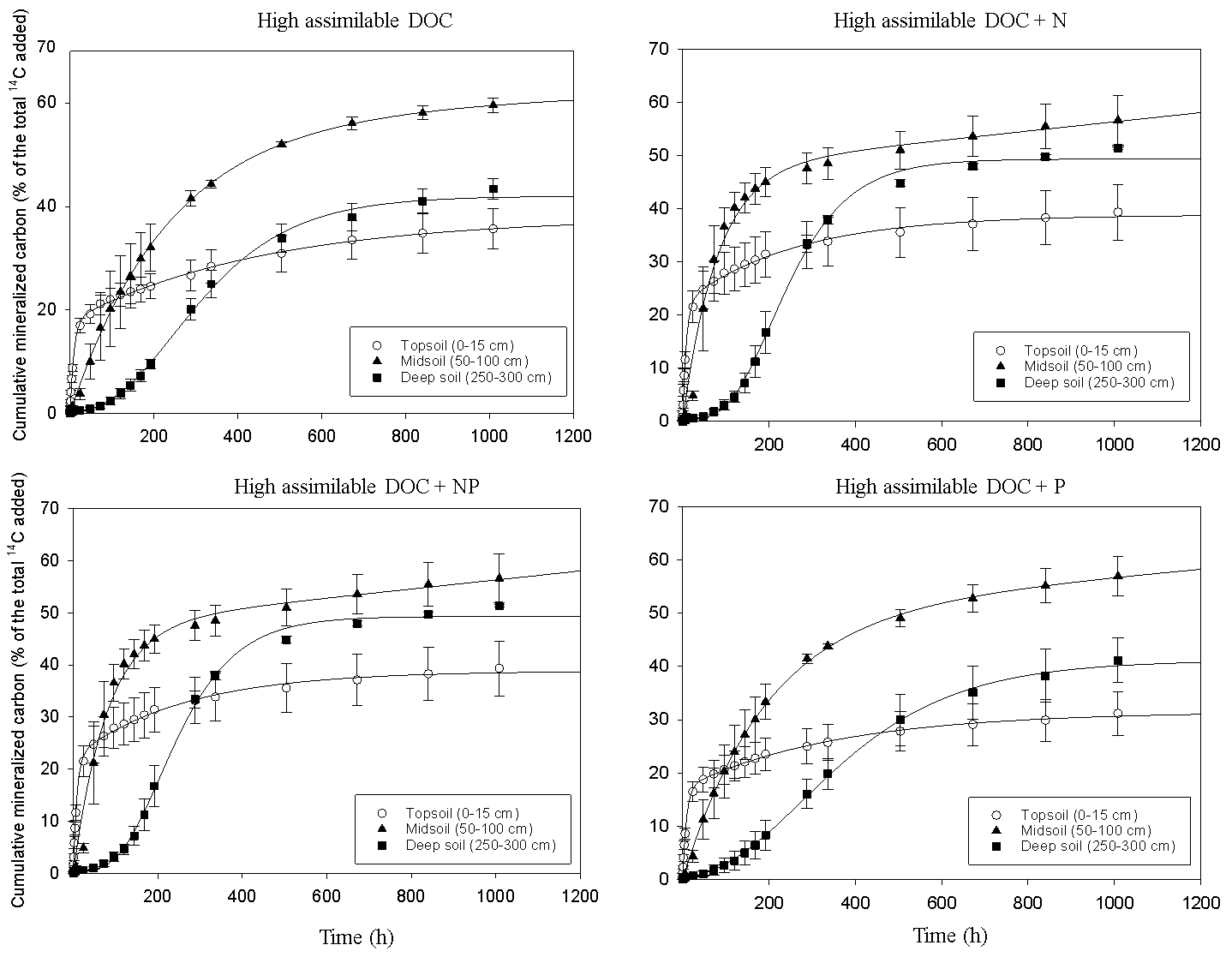
**

**Fig. S4**. Example of different microbial distance patterns as evidenced from the cumulative mineralization of substrate-C after the addition of a high dose of low molecular weight DOC either alone or in combination with N, P or N+P during a 42 d incubation at three different soil depths. The curves are only presented for distance 1 (2 m from the river) for topsoil and midsoil and distance 3 for the deepsoil in the riparian transect. Bars represent mean values (*n* = 3) ± standard errors.


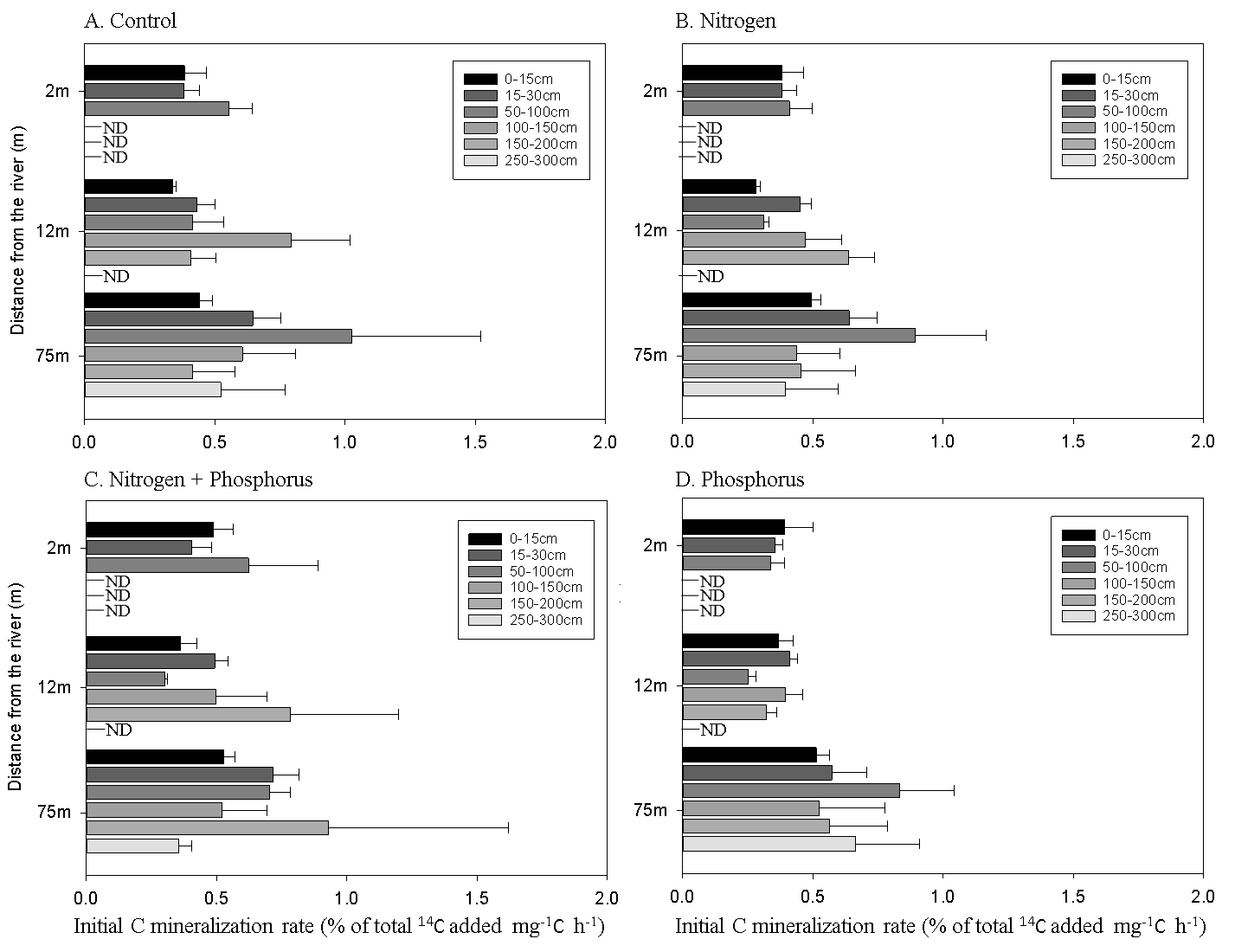


**Fig. S5.** Initial C mineralization rates (microbial C biomass normalized) measured during the initial linear phase (between 0-6 h) after the addition of a high dose of low molecular weight DOC either alone or in combination with N, P or N+P. Values are presented for three different distances from the river (2, 12 and 75 m) and for 6 different soil depths. Bars represent mean values (*n* = 3) ± standard errors. ND refers to missing values indicate no samples due to hitting bedrock.


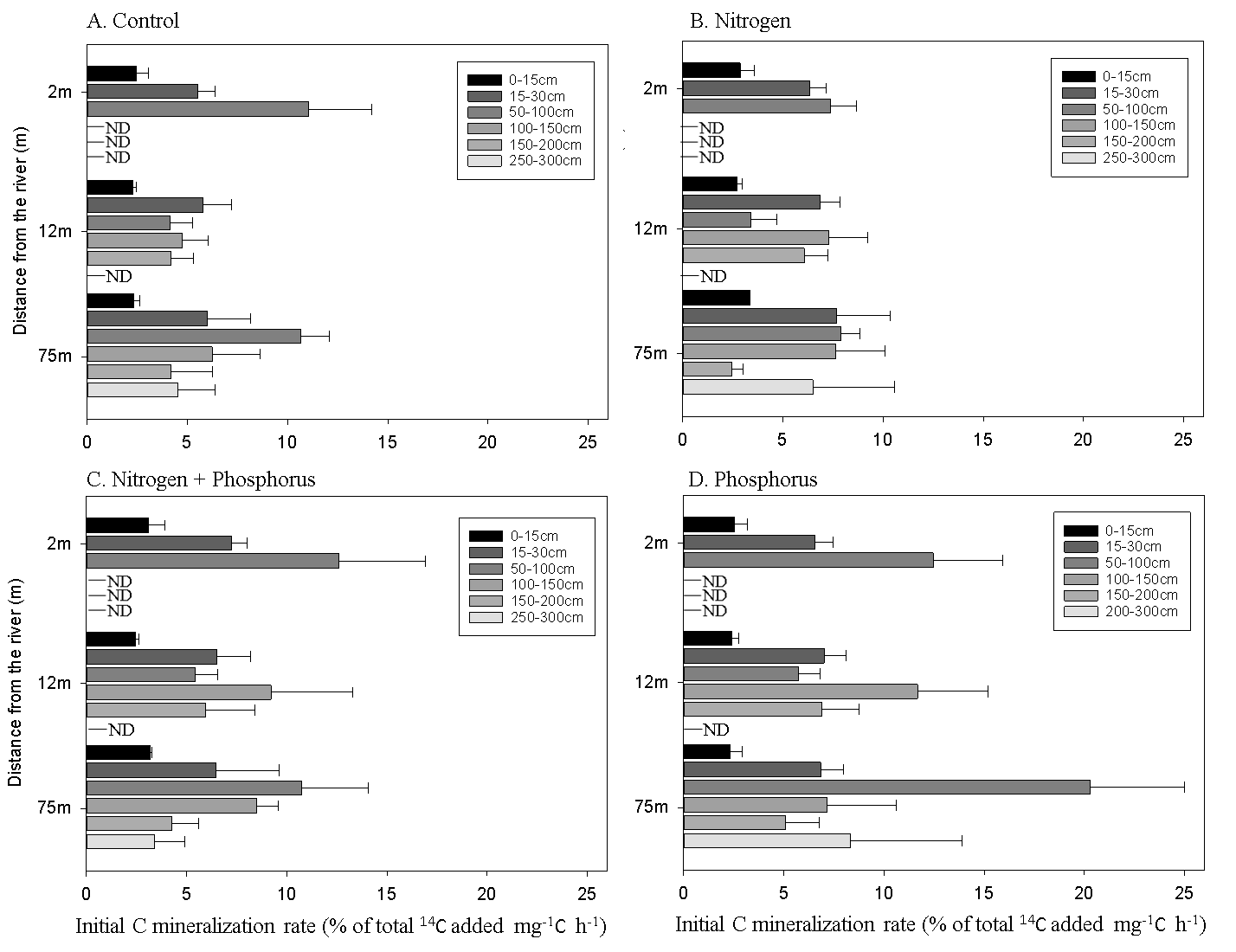


**Fig. S6.** Initial C mineralization rates (microbial C biomass normalized) measured during the initial linear phase (between 0-6 h) after the application of a low dose of low molecular weight DOC either alone or in combination with N, P or N+P. Values are presented for three different distances from the river (2, 12 and 75 m) and for 6 different soil depths. Bars represent mean values (*n* = 3) ± standard errors. ND refers to missing values indicate no samples due to hitting bedrock.


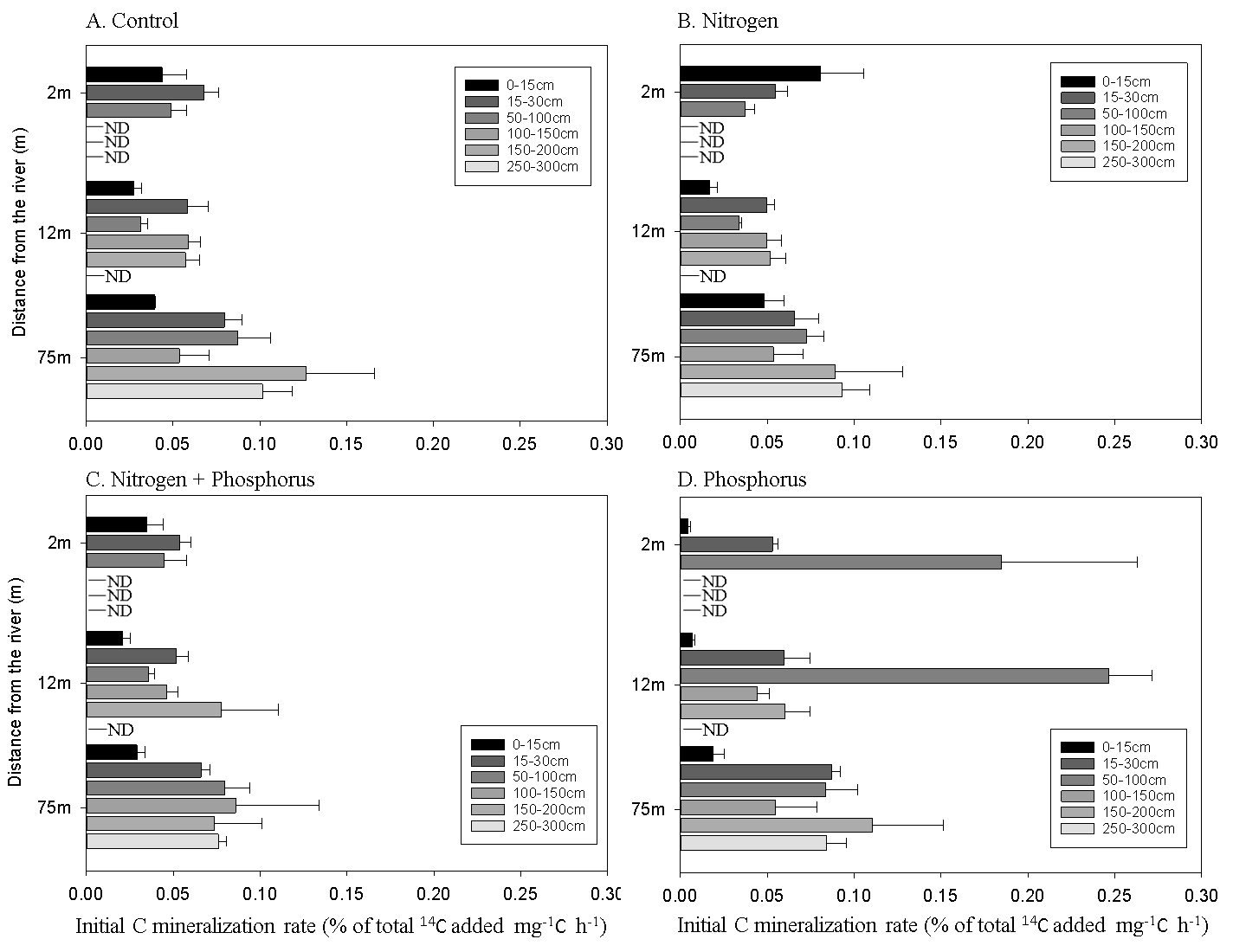


**Fig. S7.** Initial C mineralization rates (microbial C biomass normalized) measured during the initial linear phase (between 0-48 h) after the application of a medium dose of high molecular weight DOC either alone or in combination with N, P or N+P. Values are presented for three different distances from the river (2, 12 and 75 m) and for 6 different soil depths. Bars represent mean values (*n* = 3) ± standard errors. ND refers to missing values indicate no samples due to hitting bedrock.

**Table S1**. Soil physicochemical properties according to soil depth and distance from the river (distance 1, 2 m; distance 2, 12 m; distance 3, 75 m). Different upper-case letters indicate significant differences (*P* < 0.05) according to One-way ANOVA with depth as the main factor followed by a Games-Howel post-hoc test. Different lower-case letters indicate significant differences (*P* < 0.05) with respect to distance from the river according to One-way ANOVA followed by a Tukey post-hoc test. Value are means ± standard errors (*n* = 3). All the PLFA biomass values below a soil depth of 100 cm were combined due to the low abundance of organisms present. Only PLFA soil biomass up to 100 cm was included in the statistical analysis. Missing values indicate no samples due to hitting bedrock.

| **Soil property** | **Distance** | **Soil depth** | | | | | | | | | | | | | | | | | | | | | | | | |
| --- | --- | --- | --- | --- | --- | --- | --- | --- | --- | --- | --- | --- | --- | --- | --- | --- | --- | --- | --- | --- | --- | --- | --- | --- | --- | --- |
|  | **from the river** | **0-15 cm** | | | | **15-30 cm** | | | | **50-100 cm** | | | | **100-150 cm** | | | | **150-200 cm** | | | | | **250-300 cm** | | | |
| **pH** | 2 m | 5.58 | ± | 0.18 |  | 5.87 | ± | 0.19 |  | 6.16 | ± | 0.09 | ab |  |  |  |  | |  |  |  |  | |  |  |  |
|  | 12 m | 5.34 | ± | 0.20^A^ |  | 5.35 | ± | 0.23^A^ |  | 6.03 | ± | 0.05^AB^ | b | 6.48 | ± | 0.10^AB^ |  | | 7.03 | ± | 0.28^B^ |  | |  |  |  |
|  | 75 m | 5.52 | ± | 0.04^A^ |  | 5.73 | ± | 0.13^AB^ |  | 6.57 | ± | 0.14^B^ | a | 6.36 | ± | 0.29^AB^ |  | | 6.28 | ± | 0.34^AB^ |  | | 6.74 | ± | 0.32^AB^ |
| **EC**  **(µS cm^-1^)** | 2 m | 63.0 | ± | 13.1 |  | 25.2 | ± | 5.4 |  | 34.1 | ± | 14.5 |  |  |  |  |  | |  |  |  |  | |  |  |  |
|  | 12 m | 33.6 | ± | 8.5 |  | 58.5 | ± | 41.9 |  | 18.6 | ± | 1.5 |  | 34.5 | ± | 8.4 |  | | 47.7 | ± | 13.4 |  | |  |  |  |
|  | 75 m | 77.3 | ± | 44.3 |  | 28.0 | ± | 8.4 |  | 14.5 | ± | 1.12 |  | 18.7 | ± | 1.4 |  | | 20.2 | ± | 3.9 |  | | 22.1 | ± | 1.9 |
| **Moisture Content**  **(g kg^-1^ soil)** | 2 m | 296 | ± | 17^A^ |  | 240 | ± | 10^AB^ |  | 179 | ± | 19^B^ |  |  |  |  |  | |  |  |  |  | |  |  |  |
|  | 12 m | 333 | ± | 9^A^ |  | 257 | ± | 8^B^ |  | 216 | ± | 4^ABC^ |  | 133 | ± | 2^C^ |  | | 133 | ± | 29^BC^ |  | |  |  |  |
|  | 75 m | 304 | ± | 9^C^ |  | 236 | ± | 9^A^ |  | 136 | ± | 16^AB^ |  | 125 | ± | 11^B^ |  | | 110 | ± | 2^B^ |  | | 107 | ± | 4^B^ |
| **Organic matter**  **(g kg^-1^ soil)** | 2 m | 62.2 | ± | 6.3^A^ | a | 3.71 | ± | 3.1^A^ | a | 16.5 | ± | 3.4^B^ |  |  |  |  |  | |  |  |  |  | |  |  |  |
|  | 12 m | 76.7 | ± | 3.6^C^ | ab | 5.11 | ± | 1.5^A^ | ab | 25.4 | ± | 6.8^AB^ |  | 11.1 | ± | 1.3^B^ |  | | 11.2 | ± | 2.4^B^ |  | |  |  |  |
|  | 75 m | 89.4 | ± | 3.5^A^ | b | 5.61 | ± | 5.7^AB^ | b | 18.8 | ± | 1.5^B^ |  | 14.3 | ± | 0.6^B^ |  | | 12.8 | ± | 1.0^B^ |  | | 13.9 | ± | 0.7^B^ |
| **Ammonium**  **(NH_4_^+^-N)**  **(mg kg^-1^ DW soil)** | 2 m | 1.71 | ± | 0.12 | a | 1.13 | ± | 0.23 | a | 0.95 | ± | 0.33 | a |  |  |  |  | |  |  |  |  | |  |  |  |
|  | 12 m | 3.45 | ± | 0.69^A^ | b | 3.62 | ± | 1.33^AB^ | b | 0.85 | ± | 0.09^AB^ | a | 0.98 | ± | 0.21^AB^ | a | | 0.77 | ± | 0.06^B^ |  | |  |  |  |
|  | 75 m | 7.39 | ± | 1.53^AB^ | c | 4.02 | ± | 0.46^A^ | b | 0.23 | ± | 0.07^B^ | b | 0.17 | ± | 0.08^B^ | b | | 0.24 | ± | 0.13^B^ |  | | 0.32 | ± | 0.07^B^ |
| **Nitrate (NO_3_-N)**  **(mg kg^-1^ DW soil)** | 2 m | 4.08 | ± | 2.40 |  | 2.90 | ± | 1.55 |  | 2.61 | ± | 1.38 |  |  |  |  |  | |  |  |  |  | |  |  |  |
|  | 12 m | 4.33 | ± | 2.65 |  | 3.87 | ± | 3.74 |  | 0.52 | ± | 0.38 |  | 2.81 | ± | 1.51 |  | | 0.45 | ± | 0.12 |  | |  |  |  |
|  | 75 m | 1.91 | ± | 1.04 |  | 3.57 | ± | 3.02 |  | 0.90 | ± | 0.42 |  | 0.16 | ± | 0.08 |  | | 1.74 | ± | 0.93 |  | | 0.40 | ± | 0.22 |
| **P available (PO_4_-P)**  **(mg kg^-1^ DW soil)** | 2 m | 22.5 | ± | 1.93^A^ | a | 2.73 | ± | 0.91^B^ |  | 16.4 | ± | 6.14^AB^ | a |  |  |  |  | |  |  |  |  | |  |  |  |
|  | 12 m | 5.51 | ± | 1.74 | b | 1.02 | ± | 0.17 |  | 1.11 | ± | 0.31 | b | 57.1 | ± | 32.5 |  | | 41.4 | ± | 17.8 |  | |  |  |  |
|  | 75 m | 3.08 | ± | 0.29 | b | 1.10 | ± | 0.31 |  | 6.61 | ± | 1.93 | a | 8.22 | ± | 2.15 |  | | 18.9 | ± | 5.06 |  | | 80.7 | ± | 21.3 |
| **C:N ratio** | 2 m | 8.39 | ± | 3.34 |  | 4.46 | ± | 0.23 | a | 2.58 | ± | 0.59 |  |  |  |  |  | |  |  |  |  | |  |  |  |
|  | 12 m | 11.1 | ± | 1.67 |  | 6.38 | ± | 1.06 | b | 1.71 | ± | 0.34 |  | 3.08 | ± | 1.21 |  | | 4.02 | ± | 0.77 | a | |  |  |  |
|  | 75 m | 9.68 | ± | 1.58^AB^ |  | 6.44 | ± | 0.29^A^ | b | 1.39 | ± | 0.26^B^ |  | 1.09 | ± | 0.17^B^ |  | | 1.01 | ± | 0.18^B^ | b | | 0.94 | ± | 0.04^B^ |
| **Dissolved organic C**  **(mg kg^-1^ DW soil)** | 2 m | 111 | ± | 18.5^A^ | a | 73.9 | ± | 7.68^A^ | a | 20.3 | ± | 8.09^B^ |  |  |  |  |  | |  |  |  |  | |  |  |  |
|  | 12 m | 186 | ± | 3.11^A^ | ab | 110 | ± | 5.50^B^ | b | 43.4 | ± | 24.4^ABC^ |  | 14.7 | ± | 9.36^C^ |  | | 3.56 | ± | 1.34^C^ | a | |  |  |  |
|  | 75 m | 238 | ± | 23.8^A^ | b | 148 | ± | 20.2^AB^ | b | 38.3 | ± | 14.2^B^ |  | 24.4 | ± | 6.08^B^ |  | | 11.9 | ± | 2.13^B^ | b | | 5.30 | ± | 0.63^B^ |
| **Total dissolved N**  **(mg kg^-1^ DW soil)** | 2 m | 30.7 | ± | 4.28^A^ |  | 17.4 | ± | 2.04^A^ |  | 4.71 | ± | 1.74^B^ |  |  |  |  |  | |  |  |  |  | |  |  |  |
|  | 12 m | 48.5 | ± | 8.08^AB^ |  | 21.7 | ± | 2.31^A^ |  | 7.15 | ± | 4.35^AB^ |  | 3.76 | ± | 2.46^B^ |  | | 2.69 | ± | 1.03^B^ |  | |  |  |  |
|  | 75 m | 46.2 | ± | 2.01^A^ |  | 25.0 | ± | 3.25^B^ |  | 6.26 | ± | 0.67^B^ |  | 5.32 | ± | 0.92^B^ |  | | 8.34 | ± | 4.94^B^ |  | | 2.77 | ± | 0.47^B^ |
| **Microbial biomass C**  **(mg kg^-1^ DW soil)** | 2 m | 853 | ± | 258^A^ |  | 264 | ± | 44^B^ |  | 102 | ± | 18^B^ | a |  |  |  |  | |  |  |  |  | |  |  |  |
|  | 12 m | 739 | ± | 67^C^ |  | 217 | ± | 31^AB^ |  | 92.2 | ± | 3.4^A^ | a | 59.1 | ± | 4.3^B^ |  | | 52.1 | ± | 9.5^AB^ |  | |  |  |  |
|  | 75 m | 670 | ± | 23^A^ |  | 238 | ± | 47^B^ |  | 36.9 | ± | 9.8^B^ | b | 37.4 | ± | 2.2^B^ |  | | 35.8 | ± | 12.5^B^ |  | | 31.3 | ± | 2.9^B^ |
| **PLFA biomass**  **(µmol kg^-1^ soil)** | 2 m | 210 | ± | 11^A^ | a | 52.1 | ± | 7.2^B^ |  | 9.57 | ± | 1.53^C^ |  |  | | | | | | | | | | | | |
|  | 12 m | 269 | ± | 29^A^ | ab | 113 | ± | 61^AB^ |  | 5.15 | ± | 1.68^B^ |  | 3.05 ± 1.29 | | | | | | | | | | | | |
|  | 75 m | 322 | ± | 8^A^ | b | 113 | ± | 7^B^ |  | 4.53 | ± | 2.07^C^ |  |  |  |  |  |  |  |  |  |  |  |  |  |  |

**Table S2**. Maximum sorption (*S*_max_) and binding energy constant (*k*) describing the binding of inorganic P to the soil with respect to distance from the river and soil depth. *S*_max_ and *k* were estimated using the Langmuir equation fitted to experimental data (*r*^2^ > 0.9, *p* < 0.001 for all cases). Different lower-case letters indicate significant differences (*P* < 0.05) with distance from the river according to One-way ANOVA followed by a Tukey post-hoc test. Values are means ± standard errors (*n* = 3). Missing values indicate no samples due to hitting bedrock.

|  | **Distance from the river** | **Soil depth** | | | | | | | | | | | | | | | | | | | | |
| --- | --- | --- | --- | --- | --- | --- | --- | --- | --- | --- | --- | --- | --- | --- | --- | --- | --- | --- | --- | --- | --- | --- |
|  |  | **0-15 cm** | | | **15-30 cm** | | | **50-100 cm** | | | **100-150 cm** | | | **150-200 cm** | | | | **250-300 cm** | | | |  |
| **Maximum P sorption *S*_max_ (mg kg^-1^)** | 2 m | 730 | ± | 73^a^ | 646 | ± | 47^a^ | 356 | ± | 55 |  |  |  | |  |  |  | |  |  |  |  |
|  | 12 m | 1037 | ± | 37^b^ | 859 | ± | 25^b^ | 500 | ± | 306 | 303 | ± | 99 | | 268 | ± | 39 | |  |  |  |  |
|  | 75 m | 1157 | ± | 46^b^ | 976 | ± | 67^b^ | 582 | ± | 65 | 462 | ± | 41 | | 403 | ± | 26 | | 327 | ± | 25 |  |
| **Binding strength *k***  **(l kg^-1^)** | 2 m | 0.72 | ± | 0.06^a^ | 0.92 | ± | 0.14^a^ | 0.55 | ± | 0.05 |  |  |  | |  |  |  | |  |  |  |  |
|  | 12 m | 1.49 | ± | 0.16^b^ | 2.17 | ± | 0.75^a^ | 2.16 | ± | 1.19 | 1.54 | ± | 0.90 | | 0.80 | ± | 0.05 | |  |  |  |  |
|  | 75 m | 2.02 | ± | 0.13^b^ | 2.40 | ± | 0.18^b^ | 1.76 | ± | 0.43 | 1.90 | ± | 0.96 | | 1.29 | ± | 0.58 | | 0.74 | ± | 0.1 |  |

**Table S3**. Total iron concentration as a function of soil depth. Iron was measured by total reflection X-ray fluorescence (TXRF) analysis. Values represent means ± standard errors (for each sampling depth with the range 0-100 cm, *n* = 9; 100-200, *n* = 6; 250-300 cm, *n* = 3).

| **Soil depth (cm)** | **Fe (g kg^-1^ soil)** | | |
| --- | --- | --- | --- |
| 0-15 | 19.3 | ± | 0.84 |
| 15-30 | 23.3 | ± | 1.41 |
| 50-100 | 26.2 | ± | 2.79 |
| 100-150 | 27.9 | ± | 2.08 |
| 150-200 | 29.4 | ± | 0.98 |
| 250-300 | 55.0 | ± | 5.85 |
